# Surrogate modeling and control of medical digital twins

**Published:** 2024-05-20

**Authors:** Luis L. Fonseca, Lucas Böttcher, Borna Mehrad, Reinhard C. Laubenbacher

**Affiliations:** 1 Laboratory for Systems Medicine, Department of Medicine, University of Florida, Gainesville, FL, USA.; 2 Department of Computational Science and Philosophy, Frankfurt School of Finance and Management, 60322 Frankfurt am Main, Germany.

## Abstract

The vision of personalized medicine is to identify interventions that maintain or restore a person’s health based on their individual biology. Medical digital twins, computational models that integrate a wide range of health-related data about a person and can be dynamically updated, are a key technology that can help guide medical decisions. Such medical digital twin models can be high-dimensional, multi-scale, and stochastic. To be practical for healthcare applications, they often need to be simplified into low-dimensional surrogate models that can be used for optimal design of interventions. This paper introduces surrogate modeling algorithms for the purpose of optimal control applications. As a use case, we focus on agent-based models (ABMs), a common model type in biomedicine for which there are no readily available optimal control algorithms. By deriving surrogate models that are based on systems of ordinary differential equations, we show how optimal control methods can be employed to compute effective interventions, which can then be lifted back to a given ABM. The relevance of the methods introduced here extends beyond medical digital twins to other complex dynamical systems.

## Author Summary

Our work enables the efficient control of biomedical and related digital twins that are based on agent-based models (ABMs), with potential applications to the optimization of therapeutic interventions. Agent-based models, also known as individual-based models, are a class of computational stochastic models in which an agent’s behavior is modeled by rules rather than by equations. This property makes them well suited for modeling biological systems for which detailed *in vivo* kinetic information is unavailable, such as many aspects of the human immune system. However, since ABMs are not equation-based, conventional optimization approaches, sensitivity analyses, and optimal control methods are inapplicable or computationally infeasible. One way to overcome this issue is to approximate a given ABM using an ordinary differential equation (ODE) surrogate model for this purpose. In this work, we propose several types of ODE surrogate models and show how to employ them to approximate ABMs for the purpose of solving optimal control problems. Most treatment optimization tasks in medicine can be formulated as optimal control problems. Hence, the approaches presented here will likely be useful in applications involving ABM-based medical digital twins.

## Introduction

The promise of personalized medicine is to determine interventions for maintaining or restoring the health of a person based on their individual biology. A key technology to realize this promise is the use of high-fidelity computational models that are calibrated to an individual patient and help guide optimal interventions to restore or maintain health. If the models can be dynamically updated to reflect changes in a person’s health status, they fall into the category of *medical digital twins*. While there are some examples of currently used medical digital twins (see, e.g., [1]), there are many obstacles to be overcome to their development at scale.

Medical digital twins often need to capture mechanisms across different spatial and temporal scales; for instance, the mode of action of many drugs is intracellular, while their effects manifest themselves at the organ or organism scale. Depending on the application, e.g., immune system applications, digital twins may also need to account for stochastic effects. As a result, high-fidelity medical digital twins can require high-dimensional, multi-scale, hybrid, stochastic, dynamic computational models at their core. This requirement poses two problems: First, optimal control theory methods, well-developed for ordinary differential equation models, are not generally applicable to complex hybrid models, leaving only *ad hoc* methods for the identification of optimal interventions. Second, high-dimensional complex models of this kind pose computational challenges to simulation, optimization, and control tasks, and they do not readily lend themselves to available data assimilation methods for dynamic calibration to new patient data. These problems have been encountered in digital twin research beyond medicine, and there are no general solutions available at this time, as detailed in the 2023 report *Fundamental Research Gaps for Digital Twins*, prepared by the National Academies of Engineering, Science, and Medicine [2].

While physics-based systems of equations provide useful descriptions of many natural phenomena, certain aspects of human biology, such as the immune system, are more effectively represented by alternative model types like agent-based models (ABMs) [3,4]. These models, characterized by rule-based dynamics, are often employed to simulate complex and spatially heterogeneous processes. To address the challenges described above, we propose a surrogate modeling solution for control problems associated with ABMs. The basic idea is as follows. *For a given control problem**, we construct a surrogate model consisting of a system of ordinary differential equations (ODEs) for which optimal control methods are readily applicable. This surrogate model can then be used as the computational core of a digital twin in place of the original problem.* This strategy was proposed initially in [5]. Here, we further develop and formalize this control approach, and present its first empirical application to control ABMs. This paper does not address the problem of personalizing a model to a given patient using patient-derived time course data. Even for ODE models, this poses formidable challenges that are beyond the scope of this study.

### Control

The concept of control is central to medicine, for example in containing the spread of a pathogen using antibiotics, regulating blood sugar levels in diabetic patients, or managing high blood pressure [6–8]. In such settings, it is often required to not only determine how to achieve control (*i.e*., treat a specific condition), but also to do so optimally without over- or undershooting, and effectively minimizing treatment side effects and costs while ensuring the desired outcomes. To illustrate the need for techniques such as those presented here, in the context of medical digital twin applications, is the treatment of patients suffering from pneumonia in an Intensive Care Unit (ICU). A computational model underlying a pneumonia digital twin would capture the immune response to the pathogen in the lungs, e.g., a more comprehensive version of the model in [9]. It is a stochastic multiscale model, with an ABM at the tissue level, Boolean network models at the intracellular scale, and partial differential equations (PDEs) for diffusion of substances. This model could be personalized to the specific pneumonia patient using data assimilation methods, applied to a mixture of data collected from the patient over time and outcome data from a suitably chosen reference patient cohort. This personalized model can then be used to simulate the effect of different possible interventions at a given time point, such as the determination and duration of administration of antibiotics, maybe in combination with other drugs. The techniques in this paper can then be used to develop appropriate surrogate models for treatment optimization.

Most engineered systems can be described using physics-based ODE models, allowing for the application of well-established optimal control methods[6,10]. However, biomedical control problems present unique hurdles to this approach. Human biology exhibits significantly greater variability across individuals [11–14] compared to engineered systems. Additionally, many systems crucial for human health, such as the immune system or the human microbiome, encompass highly diverse cell populations [15]. These systems are characterized by high-dimensional dynamic processes that go beyond the scope of representation achievable with a relatively small number of ODEs, as is typical for engineered systems. The development of mathematical and computational models of complex biomedical dynamics is still in its infancy and demands further research [9,16–21]. In this paper, we introduce an efficient approach to model-based control that addresses some of these challenges. While our primary focus is on applications to medical digital twins in particular, the tools we present have broad applicability, extending, for instance, to ecological models like predator-prey systems [22–24].

### Agent-based models

A key modeling paradigm for parts of human biology that are not easily described through an equation-based approach is the class of *agent-based models* (ABMs), or *individual-based models* [9,19–21,25]. These are computational models that allow the explicit representation of entities (*i.e*., agents) such as cells in a tissue or microbes in a biofilm [26–31]. Agents follow a set of typically stochastic rules to navigate a heterogeneous spatial environment and engage in interactions with one another. This class of models is intuitive and easy to implement and is hence well-suited to be used by domain experts without extensive knowledge of computational modeling. ABMs have been used in a wide range of medical applications, from studies of the immune system, tumor growth and treatment to drug development [9,19–21]. A major drawback of ABMs, however, is that they are typically not equation-based and are therefore not amenable to standard mathematical analysis, leaving simulation as the primary approach. Moreover, there is a lack of well-established mathematical tools for model-based control.

An *et al.* [5] proposed a general approach to finding effective controls for ABMs by approximating them with a system of ODEs. This approximation is designed to capture the fundamental aspects of the control problem, even if it might not precisely match an ABM’s dynamics under different initial conditions. In essence, this method treats an ABM as a high-fidelity model of the real world, while the employed ODE system acts as a surrogate model specific to the control problem at hand. In this way, one can build a comprehensive high-resolution model of human biology, and then create a simpler surrogate model for a given control problem. For digital twin applications, this approach is more appropriate than creating individual models for each new control application. It also avoids the drawback of having to decide *a priori* what part of a patient’s biology is relevant to the current control application.

**Fig 1.**
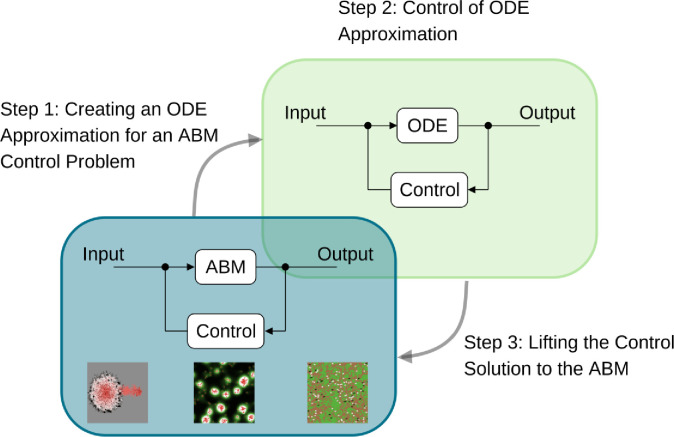
Summary of the key steps involved in using ODE surrogate models for control. For an ABM associated with a control problem, we first create an ODE surrogate (Step 1). Next, we apply control techniques to the ODE system (Step 2) before lifting the control solution back to the original ABM (Step 3). The snapshots in the ABM panel depict simulations performed in NetLogo[34] of tumor[35], slime mold[36], and wolf sheep predation models[37].

We sought to implement this strategy and provide a detailed application in two specific use cases. In doing so, we combine the strengths of both ABM and ODE models: the intuitive computational modeling of spatially heterogeneous, rule-driven stochastic systems with the effectiveness of mathematical ODE control approaches. We summarize the different steps of the proposed control approach in Fig 1. Our method’s broad applicability stems from its ability to (i) leverage the rich repertoire of modeling features of an ABM, (ii) closely approximate mechanistic features, when available, ensuring alignment with the underlying model mechanisms, and (iii) employ techniques to approximate an ABM that is near a relevant steady state for control purposes. While the ABMs we consider here are relatively simple compared to many ABMs appearing in realistic applications, they describe fundamental multi-species and biochemical dynamics that can be expected to arise in many applications of medical digital twins. Thus, they present many of the fundamental challenges to be addressed without obscuring them with biological and computational complexity. The steps described in the following sections can be applied to digital twin systems or any other stochastic model for which only forward simulations are feasible.

Several fundamental differences between ABMs and ODEs need to be considered. First, almost all ABMs are stochastic models, whereas ODE models are deterministic. Second, ABMs usually account for heterogeneous spatial features, including structured environments that agents navigate and a heterogeneous population of agents within this environment. As a result, an ODE approximation of an ABM is unlikely to fully capture ABM dynamics to a degree that it can serve as a universally reliable approximation for general model simulation. In essence, ABMs are characterized by microstates at the agent level that generate emergent dynamics of population-level macrostates, whereas ODE models can only capture aggregate macrostates. ODE approximations can, nevertheless, approximate the solution of a given control problem within a restricted domain. Additionally, previous studies have demonstrated that ODE surrogate models of complex networked dynamical systems can provide accurate descriptions of their evolution in certain cases [32,33].

The diversity in structure among ABMs poses a significant challenge for any general approximation method, including ours, rendering the formulation and implementation of a general algorithm difficult. The fact that there are no generally accepted implementation standards available for ABMs, contrary to the Systems Biology Markup Language (SBML) standard for ODEs [38–41], complicates this approach further. While the text-based Overview, Design concepts, Details (ODD) protocol [42,43] provides a useful framework for describing ABMs, it leaves many ambiguities in how to map such a description to an actual implementation [44].

### Surrogate Modeling

Surrogate models, also known as metamodels, proxy models, or emulators[45,46], are a class of models developed to approximate another model, often called the high-fidelity model. The former are usually simplified models, which allow one to predict the evolution of the latter using fewer computational resources, resulting in a tradeoff between accuracy and speed. Additionally, surrogate models are also commonly employed for performing sensitivity analysis, uncertainty quantification, risk analysis, and optimization. As pointed out by Bahrami *et al.* [45], surrogate modeling does not replace the development of high-fidelity, high-accuracy models. The latter are an important part of modeling large, complex, multiscale systems. Surrogate models serve mainly as supplementary tools for analyzing existing high-fidelity, high-accuracy models. They enable specific analyses that would otherwise be impractical (*e.g*., optimal control) or resource-intensive (*e.g*., sensitivity analysis and optimization).

In their review Bahrami *et al.* [45] describe a classification scheme of proxy models, which is valuable for characterizing the surrogate models developed here. In order to enable the application of optimal control methods within a dynamic ABM, two key requirements must be met: (i) the presence of a dynamic surrogate model, and (ii) compatibility with a formalism supporting optimal control theory. These criteria are both satisfied by employing an ODE-based surrogate model. Some authors [45,47] categorize surrogate models as either physics-based or black-box. For the purpose of this paper, we suggest using a spectrum of surrogate models. If enough information about a high-fidelity ABM is available, one can employ a mechanistic (physics-based) approximation. If limited information is available about a given ABM, or if an automatically generated surrogate model is preferred, we propose a technique for constructing a black-box model, such as an S-system[48,49]. However, if it is feasible to infer the flow of mass through the components of the high-fidelity ABM, one may consider using an ODE defined with a stoichiometric matrix that regulates this mass flow. Alternatively, the processes within the ODE could be approximated using general functions, such as power laws. This approach sidesteps the more challenging task of mechanistically approximating the processes. Hence, the resulting surrogate model is only semi-mechanistic, or what is sometimes referred to as a grey-box model.

After formulating the surrogate models, we propose to parameterize them through fitting, using maximum likelihood estimation against datasets produced by simulating the high-fidelity model. While various methods exist for dataset generation and fitting [47], we recommend selecting one based on the specific problem. We chose several datasets generated with diverse initial conditions and varying levels of control. This strategy ensures that the surrogate model effectively approximates the original model across a broad range of values in both the state variables and the control parameters.

After describing the algorithm in the Materials and Methods, we apply it to two ABMs. The first is a well-studied multi-species ABM from ecology, a predator-prey model with a resource component, implemented as wolves and sheep, with grass as the resource [37]. This model is implemented in the popular modeling platform Netlogo [34]. We have chosen this application for two reasons: (1) to demonstrate the power and versatility of our method without the technical challenges of a more complex ABM, and (2) because this model type is broadly used in biomedicine; for instance, most in-host infection models are essentially predator-prey models, where immune cells behave as predators and pathogenic cells as prey. The second ABM is a model of a metabolic network with 5 metabolites, 4 reactions and two regulatory interactions. This second model is larger and more complex than the first. It has both microstates and macrostates. The microstates are characterized by the elementary reactions between metabolites and enzymatic complexes, and the macrostates are given by Michaelis–Menten approximations. Many biological and medical processes are modeled using Michaelian or Monod processes. This second model was created with the objective of having a challenging test case for power law–based surrogate models, both in structure and initial conditions. We considered this step necessary to pinpoint the limitations of these surrogate models and explore potential alternative solutions.

## MATERIALS AND METHODS.

### Algorithm Input.

The starting point of the algorithm is a control problem derived from a medical application, using a digital twin with an underlying ABM. The ABM may be specified at different levels of detail, ranging from a detailed description and source code following an ODD protocol [42,43,50] to just an executable file. Relevant control problems include scenarios where a single control input is needed to transition the model from one steady state to another, as well as situations requiring feedback control to keep one or more state variables within a specified range.

### Mapping of ABM features to ODE features.

Rather than aiming to replicate all key ABM features in an ODE approximation, a more effective strategy is often to capture only the most critical features of an ABM relevant to the specific control problem. This process leads to a reduced ODE surrogate model that can be used for optimal control purposes. As mentioned earlier and further detailed in the Discussion section, there are several reasons why it might be useful to initially construct a complex high-fidelity ABM and then proceed to model this ABM with a coarser surrogate ODE model, rather than creating a simplified ODE model directly from the beginning. The detailed steps in this surrogate modeling process depend to some extent on the nature of the ABM. In many cases, it might be possible to use sensitivity analysis to determine the ABM parameters and state variables most important to the control problem and represent them explicitly in the ODE, while aggregating or eliminating the others.

**Fig 2.**
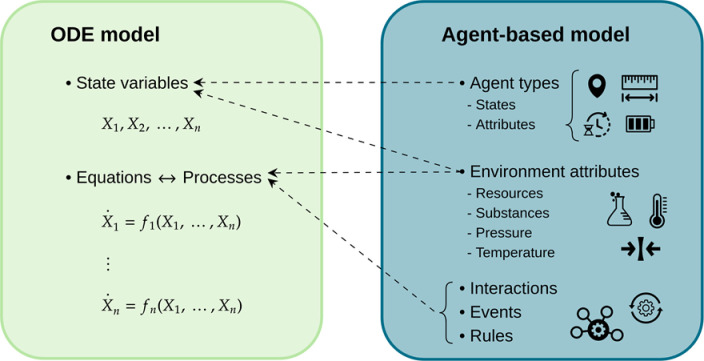
Correspondence between ABM and ODE model components. The aggregation of agents by type or attributes characterizes the state variables of an ODE approximation. Similarly, all interactions, events, and rules in the ABM characterize the processes of an ODE model. Depending on the ABM’s structure, its environment can be transformed into state variables or contribute to the processes of an ODE approximation.

Ordinary differential equations and ABMs are distinct modeling frameworks. We therefore first establish a correspondence between their respective model components (Fig 2).

Ordinary differential equations are defined by a set of state variables, each of which changes according to an equation that sums up the different processes that affect the state variable in question. On the other hand, ABMs consist of several different components. The entities that collectively make up the state of an ABM include “agents” (or “individuals”) that can take on values from a set of internal states and are equipped with a set of rules that govern their interactions with each other and with the environment in which they operate, resulting in changes of their internal states and spatial position. Other components may include properties of the spatial environment. Additionally, ABMs encompass a description of processes governed by agent and environment rules, along with scheduling details for these processes and information regarding their respective time scales. The ODD protocol [42,43,50] provides a systematic way to organize this information.

As the first step in the algorithm, we map all ABM components determined to be relevant to the optimal control problem to an ODE framework (Fig 2). This is a key step, and there are usually many choices of how to do this. Recall that the goal of the proposed approximation method is to solve a given ABM control problem by mapping it to an ODE model, use the rich set of control approaches available for such models, and then map the solution back to the ABM. The goal is not to identify the best ODE representation capable of reproducing the general dynamics of an ABM, which might require very different modeling choices.

The algorithm is divided into cases according to the information available about the ABM. The more information that can be used, the better the ODE approximation will generally be for solving the control problem. The best scenario is complete knowledge of the model components, that is, an ODD protocol for the model, which allows the construction of an ODE model based on key mechanistic features. At the other end of the spectrum is the ABM model as an executable program from which simulation trajectories can be obtained, but no mechanistic information is available.

### CASE 1: Information about ABM mechanisms is available.

#### Input:

Information from an ABM that enables the determination of the exact functional form and all terms of each differential equation (including the underlying stoichiometric matrix) in an ODE surrogate model.

If enough mechanistic information about an ABM is available, then it is possible to construct an ODE approximation that captures key mechanistic details relevant to the control problem, potentially leading to a better-performing approximation. For such a mechanistic approximation, we require information about the processes in the ABM to determine the complete model structure, (i) the terms appearing in each differential equation describing a state variable, and (ii) the exact functional form of each of these terms. Having access to a detailed description of the rules of an ABM allows us to determine the terms in the differential equations describing the evolution of each state variable in an ODE surrogate model. In the case of a biochemical network model, this would be the stoichiometric matrix of the model.

**Step 1:** *Identify the state variables of the ODE*. Any agent state that separately from all others modulates a process of interest or is changed by a process of interest, must be represented by its own state variable. Additionally, if control will be applied to a given agent type or state, then this agent will also have to be explicitly included as a state variable. Similarly, any agent type or state that will be used as a control signal independently of all others will have to be explicitly included as a state variable. If several agents always modulate the same processes in the same way, then it might be possible to aggregate or lump these agents into a single state variable. For example, if a certain agent type modulates a process independent of its position, then position-related information may be discarded, otherwise agents may need to be aggregated into position-dependent states.

**Step 2:** *Determine the stoichiometric matrix*. This requires identifying which state variables are changed by each of the processes (interactions or events) and specifying the sign of the effect: positive if the process leads to an increase of the state variable and negative if it leads to a decrease. Once all state variables and processes have been identified, as well as the dependencies of variables on processes, then the system may be written as

(1) $$\frac{dX}{dt}=M\cdot F$$

where $X=\left(X_{1},\ldots ,X_{n}\right)$ is the vector of all n state variables, $F=\left(F_{1},\ldots ,F_{m}\right)$ is the vector of all m processes, and $M=\left(a_{ij}\right)$ is the n×m stochiometric matrix. The stoichiometric matrix is usually sparse, with a non-zero entry $a_{ij}$ of either 1 or −1 in position (i,j) indicating that the process $F_{j}$ affects the state variable $X_{i}$ positively or negatively, respectively.

**Step 3.** *Determine the functional form of the ODE process terms*. This is achieved by analyzing the nature of the processes underlying the interactions or rules of an ABM. In many cases, the processes have well-understood characteristics, such as mass action kinetics [51–55], typical of biochemical systems, epidemiological systems like SIR [56], ecological systems like Lotka–Volterra models [57,58] and related population-based models; Henri–Michaelis–Menten kinetics [59–61] used in enzymatic reaction models; or Monod kinetics [62] used in bioreactor models. These can be incorporated into an ODE surrogate model in their standard functional forms. If a process does not fall into one of these categories, there are several possible steps to identify an appropriate form of the corresponding ODE term, otherwise a generic form may be used as described in Case 2 below.

**Step 4**: *Approximate the control terms*. This is done by identifying a continuous representation of the given control problem and implementing it in the ODE surrogate model. This may be as straightforward as adding a standard control term ‘*Bu*’ to the end of the right-hand side of the appropriate state-variable equation(s) or it might require adding control to a process formulation.

**Step 5:** *Parametrize the ODE model.* Before the ODE model can be used to find the solution for the ABM control problem, its parameters need to be estimated so that the ODE model approximates the ABM dynamics. This is done by optimizing the parameters of the ODE model against a collection of ABM simulation trajectories. Given that ABMs are often stochastic, several simulation runs for the same initialization should be averaged. The range of initializations should be chosen such that the entire domain of interest of the ABM is covered with trajectories[47]. Additionally, the ODE model should also be trained with trajectories for which control is exerted on each of the control variables, thus ensuring that the ODE model is a good approximation of the ABM in its uncontrolled state and under different levels of control. The level or magnitude of control used should be more than what is expected to be the optimal solution of the ABM control problem, which ensures that solving the control problem in the ODE model is an interpolation rather than an extrapolation problem. Initial guesses of the parameters may be obtained using the time course slope method [63,64], or model-specific parameter optimization methods, as is the case for Lotka–Volterra models [65]. We compute parameter estimates $\overset{ˆ}{p}$ using the least square method

(Eq. 2) $$\overset{ˆ}{p}=\underset{p}{argmin}(∥Y(u)-X(p,u){∥}^{2}),$$

where p is the vector of all parameters of the ODE model, u is the vector of all control parameters, Y the vector of all timepoints over all trajectories of the ABM, and X is the vector of the corresponding timepoints values obtained from the ODE model.

**Step 6:** *Solve the ABM control problem using the ODE approximation.* Standard methods from control theory can be used to solve the optimal control problem in the ODE approximation. This solution is then lifted back to the ABM. This is done by taking into consideration that now the solution may have to be discretized or the control inputs and outputs may require rescaling, depending on how these were approximated from the ABM into the ODE model (Step 4).

### CASE 2: Information is missing about the functional form of the model processes.

#### Input:

Information to determine the stoichiometric matrix of the ODE model, but not the functional form of the processes.

If sufficient information about the functional processes in the ABM is not available or the ABM processes are too complex, then either the functional forms will have to be reverse-engineered [66] or a generic functional form should be applied. Here we opted for the latter, which can be applied in a broad context. The only difference to Case 1 comes in Step 3, which we now describe.

**Step 3:** *Choosing the functional form of the processes.* If the functional form of one or more processes could not be identified in Case 1, then these will have to be identified by other means. Here, we present a biology-informed generic functional form, the power-law model.

Unlike in physics, it is often impractical to deduce concise functional expressions describing the evolution of biological processes from fundamental principles. In some cases, it is possible to derive semi-mechanistic representations of specific processes as is the case for the Henri–Michaelis–Menten approximation [59–61]. However, this seems to be more of an exception rather than a rule. Hence, when a functional representation is needed to describe a large range of different processes associated with a given ABM, there are only two other solutions: a generic function for which no guarantee exists of its correctness, or a canonical approximation.

There exist several canonical modeling frameworks in biology (as reviewed in [67]), including Lotka–Volterra systems [57,58], biochemical systems theory (BST) [68–72] and metabolic control analysis (MCA) [73,74]. Lotka–Volterra systems have limited usefulness in the context of general ABMs since they lack the flexibility to capture non-linear processes. In MCA, lin-log functions are used to describe the vector field of an ODE system [75]. This modeling framework has been primarily employed for describing enzymatic reactions. In contrast to MCA, BST expanded from its initial area of applications that mainly involved biochemical systems to other biological fields, like biomedical applications [49,76–79]. In BST, the vector field of an ODE system is described by power laws. In BST, process $F_{i}$ is given by

(Eq. 3) $$F_{j}=\alpha_{j}\prod_{k=1}^{n}X_{k}^{g_{jk}},$$

where n is the number of state variables $X_{k}$ affecting the process $F_{j},a_{j}\in R^{+}$ is the rate constant, and $g_{jk}\in R$ are the kinetic orders. In BST, kinetic orders are real-valued parameters that express the dependence of $F_{j}$ on $X_{k}$. If a state variable has a positive effect on the process, the kinetic order is positive. State variables that inhibit a process have negative kinetic orders. Likewise, a state variable that does not affect a process will have a kinetic order of zero. If the dependency of the processes on state variables are not known, it is possible to just assume that all state variables of the system may regulate each of the processes of a system. Variable selection is then left to the optimization step (see Step 5 in Case 1). Otherwise, if some state variables are known not to be involved in certain processes, then the corresponding kinetic orders should be fixed to zero, thus reducing the number of parameters to be optimized.

### CASE 3. Control at a Steady State Without Information About ODEs.

Some ABMs exhibit specific regions in state space towards which trajectories (or averaged trajectories) tend to converge. This behavior is similar to stable steady states arising in ODEs. Unlike ODEs, however, stochastic ABMs usually do not converge towards a steady state. If an ABM control problem involves a steady state, and we aim to approximate the ABM in its vicinity, then we may use a linear approximation, essentially a first-order Taylor approximation:

(Eq. 4) $$\frac{dX}{dt}=J\cdot \left(X-X_{0}\right).$$

When employing a steady-state approximation, it is important to verify that the ODE model is a good approximation of the ABM within the considered domain. Furthermore, the ODE approximation should be compatible with any control-induced displacements of the model trajectories. If the range of validity is too limited, then a second-order approximation may be used instead. That is,

(Eq. 5) $$\frac{dX_{i}}{dt}=\sum_{j=1}nJ_{ij}\left[X_{j}-{\left(X_{0}\right)}_{j}\right]+\frac{1}{2}{\left(X-X_{0}\right)}^{T}\cdot H_{i}\cdot \left(X-X_{0}\right),$$

where X is the vector of state variables, $X_{0}$ the steady state of the ABM, J the Jacobian matrix, and $H_{i}$ the Hessian matrices of each differential equation i=1,2,…,n.

When choosing between low and high-order approximations, it is important to consider that the latter, while potentially more accurate, involve a greater number of parameters. A larger number of parameters can lead to increased complexity in generating the approximation and may require a larger dataset. While a first-order approximation will have $n^{2}$ parameters, a second-order approximation will have $½\left(3n^{2}+n^{3}\right)$ parameters, where n is the number of state variables. Additionally, in second-order approximations, the corresponding ODE model may exhibit other steady states, and when control is exerted, the ODE system may be driven to or away from these regions. If the original ABM does not show evidence of any other steady states, then the solution is to select an ODE model during parameter estimation that does not have any other roots within the region of interest of the control problem.

**Step 1:** *Identify the state variables*. As in Cases 1 and 2, agent types or states have to be aggregated into state variables. However, since this approximation is not mechanistic, it may even be possible to represent only a subset of the agents as state variables and still achieve a good approximation. Because there are no mechanistic functions being approximated, the primary factor determining how agents are mapped to state variables will be the underlying control problem. Therefore, any agent state that is distinct from all others, serving as an input to the control problem or having control exerted on it, must be represented by a separate state variable.

**Step 2:** *Determine the steady state*. Using several simulations of the ABM with different initializations and averaging the region of convergence of all trajectories will yield the steady state of the ABM ($X_{0}$).

**Step 3**: *Approximate the control terms*. This is done by identifying a continuous representation of a given ABM control problem and incorporating it in the steady-state ODE approximation.

**Step 4:** *Optimize ODE Parameterization for Control.* This step is equivalent to Step 5 in Case 1.

**Step 5:** *Control of ABM using an ODE approximation.* This step is equivalent to Step 6 in Case 1.

### CASE 4. S-System approximation in the absence of any information about the ODE structure.

We finally address cases in which it is unknown how state variables affect one another – examples include the stoichiometric matrix and the functional form of the processes that regulate interactions between state variables. As explained in the Introduction, we are primarily interested in solving control problems for biomedical applications, for which the S-system approximation [48,80] is a good candidate, given that it is mathematically tractable and consists of simple functions.

In an S-system representation [48,68–72,80,81], a system of ODEs is defined with each differential equation being equal to the difference of two power-law terms (Eq. 6). The first term, which is positive, aggregates all incoming processes, while the second term, which is negative, aggregates all outgoing processes. Thus, the evolution of state variable $X_{i}$ is described by

(Eq. 6) $${\dot{X}}_{i}=\alpha_{i}\prod_{j=1}^{n}X_{j}^{g_{ij}}-\beta_{i}\prod_{j=1}^{n}X_{j}^{h_{ij}}\;\mathrm{for}\;i=1,2,\ldots ,n,$$

where, n∈N is the total number of state variables $X_{i},\alpha_{i},\beta_{i}\in R^{+}$ are the rate constants and $g_{ij},h_{ij}\in R$ are the kinetic orders.

The S-system representation (Eq. 6) models the effects that each state variable has on the positive and negative terms of the other state variables. The S-system representation does not require knowledge of the entire list of processes and interactions, as these are not explicitly modeled. Larger systems may be increasingly difficult to obtain, as the number of parameters in a system with n state variables is $2\left(n+n^{2}\right)$.

**Step 1:** *Identify the state variables of the ODE*. This step closely mirrors step 1 of Case 3. Once the list of state variables has been determined, we can write down an ODE approximation in the form of an S-system (Eq. 6).

**Step 2**: *Approximate the control terms*. This step is equivalent to Step 3 of Case 3.

**Step 3:** *Optimize ODE Parameterization for control**.* The procedure is the same as in Step 5 in Case 1. The rate constants (α and β) and kinetic orders (g and h) are estimated using datasets generated from the ABMs and the parameters of the S-system model estimated using a least square estimation method (Eq. 2).

**Step 4:** *Control of ABM using an ODE approximation*. This step is equivalent to Step 6 in Case 1.

## Results

To illustrate the algorithm and test its performance, we considered two ABM control problems to examine how well each one of the described ODE surrogate modeling methods performs in identifying appropriate optimal control solutions. The models and the control problems were chosen for their relative simplicity, aiming to illustrate the main steps of the proposed surrogate modeling and control approach.

Since future medical digital twins are anticipated to be complex hybrid ABMs, the ABMs employed in this study serve as suitable foundational models for the development of analytical tools applicable to ABMs in general and medical digital twins in particular. Moreover, the optimization of treatment within a digital twin is conceptually similar to population-level control, rendering these models valuable test cases for the development of optimal control techniques tailored to ABMs.

The first ABM is based on the sheep-wolves-grass model [37], a generalization of two-species predator-prey models [22–24,82]. This, and related models, are commonly used in ecology and systems biology to describe the interactions and competitive dynamics among different species. Its mechanistic approximation aligns with the well-established Lotka–Volterra model. The second ABM we considered was designed to be more intricate, possessing features that pose challenges for both power-law models and the S-system approximations. This model represents a simplified metabolic network with five metabolites and four enzymes. The underlying processes are stochastic discrete representations of Michaelis–Menten dynamics. Due to the increased number of variables and the presence of non-linear processes, this model exhibited a higher level of complexity compared to the first ABM.

### Sheep-wolves-grass model

We considered the sheep-wolves-grass model as implemented in NetLogo [34], and increase the “world” size and the initial number of animals by a factor of 25, resulting in a grid of size 255×255, initially containing 1,250 wolves, and 2,500 sheep. Grass was always initialized as covering 50% of the world. Further implementation details are summarized in the Supplemental Information section. Below, we provide an overview of the datasets that we generated to train the ODE surrogate models. Different datasets are associated with different initial conditions, ABM parameters, and controls. In all ABM trajectories that we used to train the ODE surrogates, we averaged 100 realizations. For the simulations without control (datasets I and II), two different sets of initial conditions were used. For the simulations with control, we used the same initial condition as in dataset I and control either grass, sheep, or wolves (datasets III, IV and V).

The datasets without control were used to train the ODE models for the baseline dynamics of the ABM, whereas the datasets in which control was exerted on each species enable us to train ODE models that incorporate the ABM’s behavior under control. The objective was to determine the constant rates at which wolves and sheep need to be removed to transition the system from its current steady state to a new one with 50% fewer wolves and 10% more sheep as compared to the original steady state. Additionally, we aimed to find a solution that minimizes the total number of animals removed. This resulted in a classical control problem with input matrix B=diag(0,1,1) and control input $u={\left[0,-\kappa_{2}Y,-\kappa_{3}Z\right]}^{T}$. Here, X is the total amount of grass, Y the total number of sheep, and Z the total number of wolves. The quantities $\kappa_{2}$ and $\kappa_{3}$ are the removal rates that we wish to determine.

We employed five different ODE approximations to identify suitable control signals in the described ABM control problem:

1. A mechanistic approximation (Case 1), resulting in a Lotka–Volterra model.
2. A generalized mass action (GMA) model, where all seven processes were modeled using power laws involving all three variables (Case 2).
3. Linear and quadratic approximations at the steady state (Case 3).
4. An S-system model (Case 4), in which each differential equation was expressed as the difference between two power-law terms involving all three variables.

We parameterized each model against either datasets I and II, or datasets I-V, to study the effect of training the ODEs on datasets containing control information. Given that the ODE surrogates were to be evaluated for their ability to identify near-optimal control solutions, incorporating control information during the training stage transforms the control problem from an extrapolation into an interpolation task.

**Fig 3.**
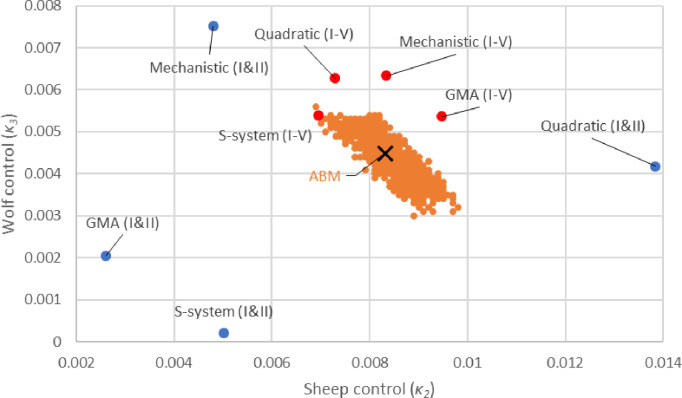
Comparison of effectiveness of different ODE surrogate models for solving the sheep-wolves-grass ABM control problem. The black cross marks the near-optimal solution ($\kappa_{2}$ = 0.83% and $\kappa_{3}$ = 0.45% per time step) for the sheep-wolves-grass ABM control problem as determined by a grid search (with a step of 0.0001 in both dimensions). Orange dots indicate suboptimal control solutions within one standard deviation from the target (a steady state with 50% fewer wolves and 10% more sheep compared to the original steady state). Blue and red dots show the control parameter values associated with the ODE surrogate models that have been calibrated against datasets I and II and datasets I-V, respectively. The best solutions were obtained for surrogate models parameterized with datasets containing control information (III-V). However, all four of these surrogate models (red dots) identified control solutions equally distant from the optimal one.

To validate the control solutions found by each ODE surrogate models, we employed a grid search to find the approximate mean optimal solution for the sheep-wolves-grass ABM control problem (black cross, Fig 3). Since the ABM never precisely reaches a steady state and instead, the three populations exhibit stochastic fluctuations around it, we also recorded all mean solutions located one standard deviation away from the target (orange dots, Fig 3). These suboptimal solutions illustrate the intrinsic level of noise present in the ABM and how it translates into the solutions of the control problem.

In Fig 3, we also show the control solutions as identified by each of the four ODE approximations, which we have parameterized either against datasets I and II (with no control information) or against datasets I-V (with control information). The linear approximation method (Case 3) delivered the worst performance in predicting the control solution and it was also the only approximation that was not able to simultaneously fit both datasets I and II. For this reason, the linear approximation was parameterized only against dataset I. All other approximations perform reasonably well at estimating the optimal solution of the ABM.

The primary factor influencing control performance was not the choice of approximations, but rather the datasets used to train the ODE models. Based on the data shown in Fig 3, we concluded that all four approximations, when parameterized against all five datasets (red dots), performed significantly better compared to when they were parameterized only against datasets I and II (blue dots). This highlights the critical importance of training the ODE surrogate models with simulations involving various levels of control to effectively solve ABM control problems. Training the ODE surrogate models on simulations where control was applied turns the estimation of the control solution from an extrapolation into an interpolation problem. We generated datasets IV and V by removing 2% of sheep and 1.5% of wolves per time step. These removal rates were set well above the approximate optimal solution of the ABM, which was identified as when 0.83% of sheep and 0.45% of wolves are concurrently removed per time step.

A possible explanation for the good performance of all four ODE surrogate models is that the mechanistic ODE model for the sheep-wolves-grass ABM aligns with a Lotka–Volterra model. This model essentially consists of a set of homogeneous second-order polynomial functions, which form a mass–action model. The other three ODE surrogates either incorporate the mechanistic model as a special case or closely resemble it. For instance, the quadratic approximation employs a set of second-order nonhomogeneous polynomial functions. The GMA approximation is constructed from a sum of power-law terms, of which mass-action serves as a special case (except for the grass growth term, which is $k_{1}\cdot X-k_{2}\cdot X^{2}$ in the mechanistic approximation and $\alpha_{1}\cdot X^{g^{11}}\cdot Y^{g12}\cdot Z^{g13}$ in the GMA approximation). The S-system is similar to the GMA, but is represented as the difference between two power-law terms, whereas the GMA model features three power-law terms in the differential equation describing the evolution of sheep.

### Metabolic pathway model

To further elucidate the differences between the different ODE surrogate models and to test their limitations, we next considered a second ABM, a metabolic pathway model for which the macroscopic mechanistic surrogate model would be given by an ODE system with Michaelis–Menten processes. In the described metabolic pathway model, we expected the ODE approximations outlined in Cases 2–4 to face difficulties in accurately representing the underlying dynamics. For example, ODE models based on power laws cannot capture saturation effects, which are characteristic of Michaelis–Menten processes. Additionally, S-system models are not good at modeling divergent and convergent pathways. The complexity of the considered metabolic dynamics could also pose difficulties for accurate approximation using first and second-order polynomials.

The metabolic pathway ABM included four reactions associated with five metabolites, and all reactions between enzymes, metabolites, and respective complexes were modeled at the elementary level (microscale). There were two agent types (metabolites and enzymatic complexes), five metabolites, four enzymes, and 12 enzyme-metabolite complexes. Metabolites moved ten times faster than enzymes or complexes, and whenever a metabolite is close to an enzyme or complex to which it may be bound, there was a probability that it may bind. Complexes could, at any time point, dissociate into their components. Enzymes formed complexes with their respective substrates, products, and regulators. Finally, enzymes complexed with their respective substrate could undergo catalysis and become a complex between the enzyme and the product. All four enzymatic reactions were modeled as irreversible.

Two collections of datasets were generated from this ABM. The first collection comprised two types of simulations: dataset I involved a single simulation where most of the pathway substrate was depleted, leading to the accumulation of two products (pathway operating in batch mode); and dataset II involved a single simulation where substrate was continuously supplied at a rate of one molecule per time step, and all metabolites were removed at a rate of 0.05% per time step (pathway operating in continuous mode as if in a continuous stirred tank reactor).

The second collection consists of three datasets, each resulting from the average of 100 simulations of the ABM under the same initial conditions. Datasets III and IV are based on averaging 100 simulations using the same parameters as datasets I and II, respectively. Dataset V is obtained by averaging 100 simulations under continuous mode with substrate being continuously supplied at a rate of 0.2 molecules per time step, while all metabolites were removed at a rate of 0.05% per time-step. In all continuous mode simulations, constant vessel volume was assumed, meaning that inflow and outflow match in volume, with only metabolites exiting while enzymes and enzymatic complexes remain within the vessel.

The control problem to be solved in the metabolic pathway model was the inference of the optimal inflow of substrate S that minimizes the loss of the substrate S and maximizes the amount of the products R and T at the outflow of the reactor. Mathematically, our goal was to identify the constant $Q_{\mathrm{in}}$ that minimizes the loss function,

(Eq. 7) $$Loss(Q)=\sum_{k=1}^{N_{t}}\frac{S_{k}}{R_{k}\;+\;T_{k}},$$

during a simulation run of $N_{t}=50,000$ time-steps, where $S_{k}$ is the concentration of the supplied substrate, at time step k, and $R_{k}$ and $T_{k}$ are the corresponding concentrations of the end-products of the pathway.

To compare the ability of all proposed ODE surrogates (mechanistic, GMA, S-system, quadratic and linear) to learn effective control solutions, we optimized them against the two collections of datasets (‘I’ will denote models optimized against datasets I and II, generated with a single simulation of each condition; and ‘C’ will denote models optimized against datasets III-V, generated by averaging 100 simulations of each condition).

**Fig 4.**
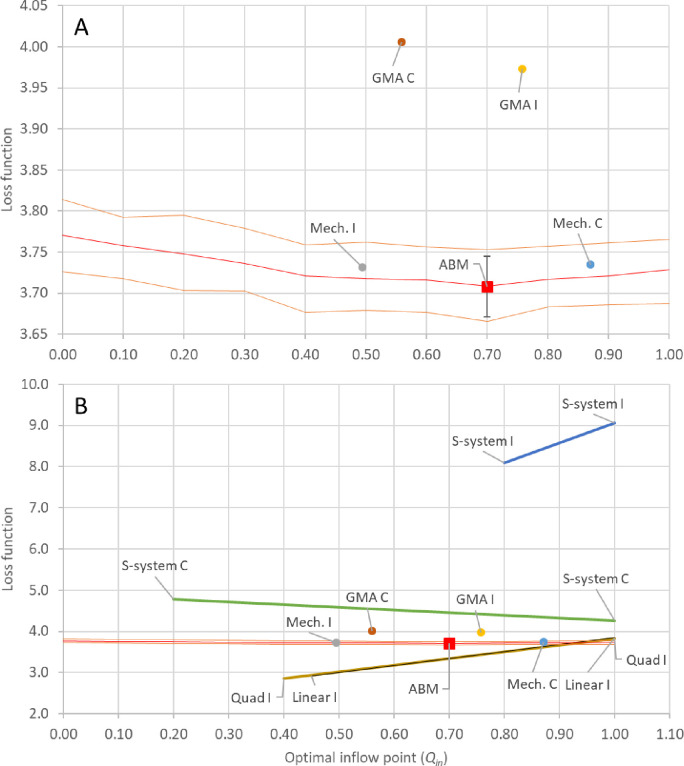
Comparison of effectiveness of different ODE surrogate models for solving the metabolic pathway ABM control problem. The red square shows the optimal inflow point and the corresponding mean loss function value as determined for the ABM by a grid search between 0 and 1.0 with a step size of 0.1, where in each step 100 simulations runs were averaged. The red line highlights the mean of each of the 100 simulation runs of the ABM and the orange line the 75% confidence band. Circles denote the predicted optimal inflow and corresponding loss function value for each ODE surrogate. ODE models that did not exhibit a minimum within the 0 to 1.0 domain have their domain of integrability shown with a line. The line depicts the range of loss function values predicted by the approximation. The S-system I performed worst, as it could only be integrated between 0.8 and 1.0, and in that range predicted loss function values between 8 and 9. While S-system C, Quad I, and Linear I, all resulted in models with a larger domain over which they could be integrated, neither had a minimum within their respective domains. GMA I was the ODE surrogate that best predicted an optimal inflow of substrate closest to the ABM and Mech. I best predicted the loss function value of the ABM at the optimal inflow point.

To evaluate the predictions of each of the ODE approximations, the optimal inflow point was estimated for the ABM by performing a grid search between 0 and 1.0 with a step of 0.1. At each step, 100 simulation runs of the ABM were performed and averaged. The best inflow of substrate was found to be 0.7 (red square, Fig 4). The red line shows the mean loss function value at each of the tested inflow points of the ABM, and the orange band highlight where 75% of the simulations reside for each inflow point.

Based on the findings presented in Fig 4, we drew the following conclusions: the GMA approximation, when parameterized against datasets I and II, performed the best in predicting the optimal substrate inflow. On the other hand, the mechanistic approximation, also parameterized against datasets I and II, performed well in estimating the loss function value. Neither of the S-system approximations displayed a minimum within the control parameter range of [0.2, 1]. Additionally, the S-systems generated against datasets I and II could only be solved between 0.8 and 1.0 due to stiffness issues. Similarly, both the quadratic and linear approximations were unable to predict an optimal inflow, as they could not be integrated across the entire domain and did not exhibit a minimum within the region where they could be integrated. However, the S-system parameterized against the averaged simulations (S-system C, derived from datasets III-V) demonstrated a notably better estimate of the loss function compared to the S-system obtained for datasets I and II (S-system I). This suggests that the S-system is more susceptible to the noise present in datasets I and II, which is reduced in the datasets resulting from averaging 100 simulations of the ABM (datasets III-V).

## Discussion

Many problems in medicine are control problems. But directly testing various control approaches, such as different treatment protocols, in the laboratory or in clinical studies is often impractical. To address this, medical digital twins offer a solution for developing effective treatments based on *in silico* treatment optimization [83–86]. The mathematical models underlying them can be diverse. One commonly used model type is ABMs, used in biomedicine to simulate complex interaction among multispecies populations. Controlling (or optimizing) ABMs is challenging, however, due to their underlying stochastic and high-dimensional dynamics and rule-based rather than equation-based structure. While traditional control methods are well-developed for ODE systems, they are not directly applicable to ABMs. To bridge the gap between standard control-theoretic approaches and ABM simulation-based methods, we proposed different types of ODE surrogate models to approximate the dynamics of a given ABM control problem. In addition to their use in control scenarios, they can also serve forecasting purposes by modeling their uncontrolled evolution. The proposed methods allow us to identify effective controls in the ODE surrogate and apply them back to an ABM. We consider this work as a small first step toward solving the general problem of surrogate modeling and optimization for medical digital twins. In summary, we developed five different approaches capable of generating ODE surrogate models from ABMs with dynamical and steady-state regimes. We employed all approximation methods on two typical ABM systems featuring Lotka–Volterra and Michaelis–Menten dynamics, which are commonly encountered in a broad spectrum of applications.

For approximations under a steady-state regime, we present two approaches: the linear and the quadratic approximations. These approaches do not require any previous knowledge of the underlying mechanisms of a given ABM control problem and they may allow one to construct approximations using only a small subset of all ABM variables. Although the linear approximation does have advantages in terms of optimal control theory, since optimal control problems associated with linear dynamical systems have analytical solutions [6–8], it was not a good approximation in either of the examples presented. A natural improvement is to employ a second-order approximation method, which we refer to as the quadratic approximation. This approximation performed better in the sheep-wolves-grass control problem than the linear approximation, but increasing the order led to the introduction of unstable steady states. Hence, it became difficult to optimize the control parameters. As a solution to this problem, we identified the best quadratic approximation that fit the ABM data and contained no other steady states within the domain of the control problem. With this additional optimization constraint, the quadratic approximation performed as well as the remaining approximations in the sheep-wolves-grass ABM.

In S-system models, each state variable is characterized by two power-law terms, one for the “inflow” and one for the “outflow” [48,80,81]. Additionally, all state variables of the system can be included in both of these terms. Given that power-law terms are linear representations of the processes in log-log space, this non-linear behavior may give it an advantage over a linear approximation. On the other hand, an S-system model will have $2\left(n+n^{2}\right)$ parameters in an *n-*dimensional ODE system, whereas a linear approximation will only have $n^{2}$ parameters. Thus, the extra non-linearity comes at the expense of more parameters. The quadratic approximation has $½\left(3n^{2}+n^{3}\right)$ parameters, which is even more than in the S-system. This makes the S-system a compromise between the linear and the quadratic approximations in terms of parameter numbers. All of these representations may gain from being parameterized with Lasso and related regularization approaches [87], which can help identify the most parsimonious model that fits the available data.

Within the mechanistic class of approaches, we considered two surrogate modeling methods: the mechanistic approximation and the GMA approximation. Among all of the ODE surrogate model methods proposed in this work, the mechanistic approach bears the closest resemblance to the equation learning approach (EQL) studied by Nardini *et al.* [88]. One key difference between the mechanistic method used in our work and the EQL method lies in how Nardini *et al.* construct a function library based on process representations inferred from a given ABM, with differential equations expressed as a linear combination of these library terms. In contrast, our approach involves utilizing an inferred stoichiometric matrix to determine which process representations have to be considered in each differential equation. In cases such as epidemiological and ecological ABMs, where both approaches are likely to result in ODEs approximated by a linear combination of polynomial terms, the resulting ODEs may seem quite similar. However, our method distinguishes itself by employing the stoichiometric matrix to determine term selection, leaving the optimization process exclusively for the refinement of process parameters. In the EQL approach, term selection and process parameters are left to the optimization process.

The GMA method represents a different paradigm. In this approach, rather than inferring the appropriate functional representation of the processes mechanistically, the functional forms are approximated by power laws and assumed to be dependent on all state variables. During the parameterization phase, a regularization method like Lasso may be used to identify a parsimonious model that is still able to capture the evolution of ABM-generated data. In GMA models, we identify not only which state variables each process depends on, but also the sign (positive for activation and negative for inhibition) and strength (the absolute value) of each dependency.

All of the approaches presented here depend on the optimization of an ODE model to ABM-generated data. Identifying an appropriate ODE surrogate model might not always be feasible. This can occur due to either the inherent limitations of the chosen surrogate model or difficulties in finding a suitable parameter set through optimization. If one expects limitations that are due to a specific optimizer choice, exploring alternative optimization techniques may provide a solution. Similar to related problems within machine learning [89], there exists a spectrum of algorithms for parameter optimization, ranging from local and usually deterministic techniques to global and stochastic ones. Furthermore, there are problem-specific approaches that require the user to understand the nature of the optimization problem and have knowledge about the most appropriate optimizers [67,80,90].

In the first example involving the sheep-wolves-grass ABM, we examined whether using training data with varying levels of control improved the predictive power of the ODE approximations. Our findings showed that optimizing ODE approximations using simulations with different control levels significantly enhanced their ability to identify suitable control signals. By training ODE approximations on ABM datasets obtained with both higher and lower levels of control relative to the true optimum, we effectively transform the problem of inferring the optimal control from an extrapolation to an interpolation task. This transformation is contingent on knowing or being able to assume or infer the domain of the control problem.

In the metabolic pathway ABM, we compared the effectiveness of averaged ABM simulations with single simulations. The results provided a more detailed picture of the performance differences between the various ODE surrogate models. For the mechanistic and GMA surrogates that provided relatively accurate estimates of the optimal control input, averaging simulations did not confer any notable advantage. In the case of the S-system surrogate, it did not perform well in estimating a suitable control signal, but it did demonstrate an improved ability to estimate the loss function when we employed averaged training data.

Among all the methods studied in this work, the GMA appears to be the most practical choice. While the mechanistic approximation is likely to yield the most accurate surrogate model, it is impractical to generate mechanistic surrogates for complex multiscale ABMs like the ones considered here and in related works (see, *e.g*., [9,20,21]). The GMA approach bypasses the need for detailed mechanistic information about the ABM, focusing solely on understanding the structure of the processes. This is a less complex task compared to capturing process representations. In the examples provided here, the GMA approach performed as effectively as the mechanistic approach (see Table 1 for a summary of the properties of the different methods).

On the other end of the spectrum lies the S-systems approach, which does not require detailed mechanistic information about a given ABM (Table 1). Instead, it requires knowledge of which agent types or states will be aggregated into each of the state variables of the surrogate model. However, in the metabolic pathway example, intentionally designed to challenge the S-system approach due to its complex structure and functional representation, the S-system did not perform well. None of the datasets used were able to generate an S-system surrogate capable of predicting the optimal control of the ABM. These findings underscore the importance of exploring approaches that fall between fully mechanistic and non-mechanistic surrogate models. Hybrid approximations may prove to be especially valuable in such cases.

In conclusion, we introduced four distinct families of approximation methods that can be employed to solve ABM control problems. We focused on ABMs with fully dynamic transients and those with stable steady states. The mechanistic approaches proposed here offer a key advantage in utilizing a stoichiometric matrix as a foundational structure for representing interactions and events within a given ABM. One major advantage of using mechanistic approximation methods is their interpretability. However, we also obtained promising results using different levels of generic ODEs. Among the approaches examined, the GMA and mechanistic methods were the most effective ones in identifying suitable control signals for a given ABM control problem.

There are several promising directions for future research. One avenue involves assessing the effectiveness of the approximation methods developed in this study on additional ABMs, particularly in the context of digital twin models integrated with patient data [20,21,83–85]. Furthermore, future investigations may focus on the application of data assimilation techniques to dynamically refine existing surrogate models as new data become available [91,92], especially in cases where ABMs are being reparametrized to account for updated patient information. Finally, it would be valuable to explore alternative ODE approximators or optimization methods focused on directly controlling specific aspects of an ABM [93–97]. These research paths will not only contribute to enhancing our ability to control ABMs but may also help develop more effective control approaches in biomedicine and related fields in general[98].

## Supplementary Material

Supplement 1

## Figures and Tables

**Table 1.** Overview of advantages and disadvantages of the different ODE metamodels.

Surrogate Model	Advantages	Disadvantages

**Mechanistic**	• Most accurate approximations. • Accurate over a wide range of the state space.	• Hard to formulate for complex ABMs. • Requires detailed mechanistic knowledge of the inner workings of the ABM. • ODEs may have a complex mathematical structure.
**GMA**	• No need for process-specific approximations; each process is formulated by a power law involving system state variables. • Determining the stoichiometric matrix is simpler than mechanistically approximating the processes. • Determining ODEs becomes straightforward once the stoichiometric matrix is known. • Can be semi-mechanistic by employing mechanistic approximations for some processes and representing others with power laws. • Small to moderate number of parameters.	• Still requires inferring the stoichiometric matrix of the ABM. • Power laws face challenges when approximating processes that reach saturation because power laws with positive kinetic orders tend to approach infinity.
**Taylor expansion at the steady state**	• With an accurate linear approximation, one can apply established control-theoretic methods for linear systems. • Straightforward to determine the ODEs.	• Unlikely to be a good approximation at lower orders and may only be accurate in specific regions of the state space. • Large number of parameters at high orders.
**S-system**	• Straightforward to determine the ODEs with a rule-based (canonical) approach. • Requires no mechanistic understanding of the ABM. • Moderate number of parameters. • Homogeneous ODEs with favorable mathematical properties, making steadystate solutions easy to obtain.	• Power laws face challenges when approximating processes that reach saturation because power laws with positive kinetic orders tend to approach infinity. • May encounter challenges when approximating systems with multiple processes affecting the same agent state.

## Data Availability

The code and datasets used in this work are available on GitHub: https://github.com/LaboratoryForSystemsMedicine/Metamodeling-and-Control-of-Medical-Digital-Twins_2024

## References

[1] Laubenbacher R, Mehrad B, Shmulevich I, Trayanova N. Digital twins in medicine. Nat Comput Sci. 2024;4: 184–191. doi:10.1038/s43588-024-00607-6 38532133 PMC11102043

[2] Engineering NA of, National Academies of Sciences Engineering, Medicine. Foundational Research Gaps and Future Directions for Digital Twins. Washington, DC: The National Academies Press; 2023. doi:10.17226/26894 39088664

[3] West J, Robertson-Tessi M, Anderson ARA. Agent-based methods facilitate integrative science in cancer. Trends Cell Biol. 2023;33: 300–311. doi:10.1016/j.tcb.2022.10.006 36404257 PMC10918696

[4] Budak M, Cicchese JM, Maiello P, Borish HJ, White AG, Chishti HB, Optimizing tuberculosis treatment efficacy: Comparing the standard regimen with Moxifloxacin-containing regimens. PLOS Comput Biol. 2023;19: e1010823. doi:10.1371/journal.pcbi.1010823 37319311 PMC10306236

[5] An G, Fitzpatrick BG, Christley S, Federico P, Kanarek A, Neilan RM, Optimization and Control of Agent-Based Models in Biology: A Perspective. Bull Math Biol. 2017;79: 63–87. doi:10.1007/s11538-016-0225-6 27826879 PMC5209420

[6] Åström KJ, Murray RM. Feedback systems: an introduction for scientists and engineers. Princeton university press; 2021.

[7] Lenhart S, Workman JT. Optimal control applied to biological models. CRC press; 2007.

[8] Swan GW. Applications of optimal control theory in biomedicine. M. Dekker New York; 1984.

[9] Ribeiro HA, Vieira LS, Scindia Y, Adhikari B, Wheeler M, Knapp A, Multi-scale mechanistic modelling of the host defence in invasive aspergillosis reveals leucocyte activation and iron acquisition as drivers of infection outcome. J R Soc Interface. 2022;19: 20210806. doi:10.1098/rsif.2021.0806 35414216 PMC9006013

[10] Ogata K. Modern Control Engineering, 5th edition. Pearson; 2009.

[11] Soto C, Bombardi RG, Kozhevnikov M, Sinkovits RS, Chen EC, Branchizio A, High frequency of shared clonotypes in human T cell receptor repertoires. Cell Rep. 2020;32. 10.1016/j.celrep.2020.107882 PMC7433715 32668251

[12] Soto C, Bombardi RG, Branchizio A, Kose N, Matta P, Sevy AM, High frequency of shared clonotypes in human B cell receptor repertoires. Nature. 2019;566: 398–402. 30760926 10.1038/s41586-019-0934-8 PMC6949180

[13] Laydon DJ, Bangham CR, Asquith B. Estimating T-cell repertoire diversity: limitations of classical estimators and a new approach. Philos Trans R Soc B Biol Sci. 2015;370: 20140291. 10.1098/rstb.2014.0291 PMC4528489 26150657

[14] Böttcher L, Wald S, Chou T. Mathematical Characterization of Private and Public Immune Receptor Sequences. Bull Math Biol. 2023;85: 1–31. 10.1007/s11538-023-01190-z PMC10501991 37707621

[15] Xu S, Böttcher L, Chou T. Diversity in biology: definitions, quantification and models. Phys Biol. 2020;17: 031001. 31899899 10.1088/1478-3975/ab6754 PMC8788892

[16] Dessalles R, D’orsogna M, Chou T. Exact steady-state distributions of multispecies birth–death–immigration processes: Effects of mutations and carrying capacity on diversity. J Stat Phys. 2018;173: 182–221.

[17] Dessalles R, Pan Y, Xia M, Maestrini D, D’Orsogna MR, Chou T. How naive T-cell clone counts are shaped by heterogeneous thymic output and homeostatic proliferation. Front Immunol. 2022;12: 735135. 35250963 10.3389/fimmu.2021.735135 PMC8891377

[18] Remien CH, Eckwright MJ, Ridenhour BJ. Structural identifiability of the generalized Lotka–Volterra model for microbiome studies. R Soc Open Sci. 2021;8: 201378. 34295510 10.1098/rsos.201378 PMC8292772

[19] Ribeiro HAL, Scindia Y, Mehrad B, Laubenbacher R. COVID-19-associated pulmonary aspergillosis in immunocompetent patients: a virtual patient cohort study. J Math Biol. 2023;87: 6. doi:10.1007/s00285-023-01940-6 37306747 PMC10258510

[20] Joslyn LR, Linderman JJ, Kirschner DE. A virtual host model of Mycobacterium tuberculosis infection identifies early immune events as predictive of infection outcomes. J Theor Biol. 2022;539: 111042. doi:10.1016/j.jtbi.2022.111042 35114195 PMC9169921

[21] Joslyn LR, Flynn JL, Kirschner DE, Linderman JJ. Concomitant immunity to M. tuberculosis infection. Sci Rep. 2022;12: 20731. doi:10.1038/s41598-022-24516-8 36456599 PMC9713124

[22] Freedman HI. Deterministic Mathematical Models in Population Ecology. Marcel Dekker Inc; 1980.

[23] Oremland M, Laubenbacher R. Optimal Harvesting for a Predator-Prey Agent-Based Model using Difference Equations. Bull Math Biol. 2015;77: 434–459. doi:10.1007/s11538-014-0060-6 25559457

[24] Colon C, Claessen D, Ghil M. Bifurcation analysis of an agent-based model for predator–prey interactions. Ecol Model. 2015;317: 93–106.

[25] An G, Cockrell RC. Agent-Based Modeling of Systemic Inflammation: A Pathway Toward Controlling Sepsis. Methods Mol Biol. 2021;2321: 231–257. doi:10.1007/978-1-0716-1488-4_20 34048021

[26] Weston B, Fogal B, Cook D, Dhurjati P. An agent-based modeling framework for evaluating hypotheses on risks for developing autism: Effects of the gut microbial environment. Med Hypotheses. 2015;84: 395–401. 25670416 10.1016/j.mehy.2015.01.027

[27] Lin C, Culver J, Weston B, Underhill E, Gorky J, Dhurjati P. GutLogo: Agent-based modeling framework to investigate spatial and temporal dynamics in the gut microbiome. PLoS One. 2018;13: e0207072. 30412640 10.1371/journal.pone.0207072 PMC6226173

[28] Bauer E, Zimmermann J, Baldini F, Thiele I, Kaleta C. BacArena: Individual-based metabolic modeling of heterogeneous microbes in complex communities. PLoS Comput Biol. 2017;13: e1005544. 28531184 10.1371/journal.pcbi.1005544 PMC5460873

[29] Becker N, Kunath J, Loh G, Blaut M. Human intestinal microbiota: characterization of a simplified and stable gnotobiotic rat model. Gut Microbes. 2011;2: 25–33. 21637015 10.4161/gmic.2.1.14651

[30] Archambault L, Koshy-Chenthittayil S, Thompson A, Dongari-Bagtzoglou A, Laubenbacher R, Mendes P. Corrected and Republished from: “Understanding Lactobacillus paracasei and Streptococcus oralis Biofilm Interactions through Agent-Based Modeling.” mSphere. 2023;8: e0065622. doi:10.1128/msphere.00656-22 36942961 PMC10187049

[31] Koshy-Chenthittayil S, Archambault L, Senthilkumar D, Laubenbacher R, Mendes P, Dongari-Bagtzoglou A. Agent Based Models of Polymicrobial Biofilms and the Microbiome—A Review. Microorganisms. 2021;9: 417. doi:10.3390/microorganisms9020417 33671308 PMC7922883

[32] Gleeson JP, Melnik S, Ward JA, Porter MA, Mucha PJ. Accuracy of mean-field theory for dynamics on real-world networks. Phys Rev E. 2012;85: 026106. 10.1103/PhysRevE.85.026106 22463278

[33] Gleeson JP. Binary-state dynamics on complex networks: Pair approximation and beyond. Phys Rev X. 2013;3: 021004.

[34] Wilensky U. NetLogo. Center for Connected Learning and Computer-Based Modeling, Northwestern University, Evanston, IL. 1999. Available: http://ccl.northwestern.edu/netlogo/

[35] Wilensky U. NetLogo Tumor model. Center for Connected Learning and Computer-Based Modeling, Northwestern University, Evanston, IL. 1997. Available: http://ccl.northwestern.edu/netlogo/models/Tumor

[36] Wilensky U. NetLogo Slime model. Center for Connected Learning and Computer-Based Modeling, Northwestern University, Evanston, IL. 1997. Available: http://ccl.northwestern.edu/netlogo/models/Slime

[37] Wilensky U. NetLogo wolf sheep predation model. Center for Connected Learning and Computer-Based Modeling, Northwestern University, Evanston, IL. 1997. Available: http://ccl.northwestern.edu/netlogo/models/WolfSheepPredation

[38] Hucka M, Finney A, Sauro HM, Bolouri H, Doyle JC, Kitano H, The systems biology markup language (SBML): a medium for representation and exchange of biochemical network models. Bioinformatics. 2003;19: 524–531. doi:10.1093/bioinformatics/btg015 12611808

[39] Hucka M, Bergmann FT, Chaouiya C, Dräger A, Hoops S, Keating SM, The Systems Biology Markup Language (SBML): Language Specification for Level 3 Version 2 Core Release 2. J Integr Bioinforma. 2019;16. doi:10.1515/jib-2019-0021 PMC6798823 31219795

[40] Keating SM, Waltemath D, König M, Zhang F, Dräger A, Chaouiya C, SBML Level 3: an extensible format for the exchange and reuse of biological models. Mol Syst Biol. 2020;16: e9110. doi:10.15252/msb.20199110 32845085 PMC8411907

[41] Smith LP, Moodie SL, Bergmann FT, Gillespie C, Keating SM, König M, Systems Biology Markup Language (SBML) Level 3 Package: Distributions, Version 1, Release 1. J Integr Bioinforma. 2020;17: 20200018. doi:10.1515/jib-2020-0018 PMC7756622 32750035

[42] Grimm V, Berger U, DeAngelis DL, Polhill JG, Giske J, Railsback SF. The ODD protocol: A review and first update. Ecol Model. 2010;221: 2760–2768. doi:10.1016/j.ecolmodel.2010.08.019

[43] Grimm V, Railsback SF, Vincenot CE, Berger U, Gallagher C, Deangelis DL, The ODD protocol for describing agent-based and other simulation models: A second update to improve clarity, replication, and structural realism. J Artif Soc Soc Simul. 2020;23. Available: http://eprints.bournemouth.ac.uk/33918/

[44] Sordo Vieira L, Laubenbacher RC. Computational models in systems biology: standards, dissemination, and best practices. Curr Opin Biotechnol. 2022;75: 102702. doi:10.1016/j.copbio.2022.102702 35217296 PMC9177621

[45] Bahrami P, Sahari Moghaddam F, James LA. A Review of Proxy Modeling Highlighting Applications for Reservoir Engineering. Energies. 2022;15: 5247. doi:10.3390/en15145247

[46] Degeling K, IJzerman MJ, Koffijberg H. A scoping review of metamodeling applications and opportunities for advanced health economic analyses. Expert Rev Pharmacoecon Outcomes Res. 2019 [cited 18 Apr 2024]. Available: 10.1080/14737167.2019.1548279 30426801

[47] Ahmed MYM, Qin N. Surrogate-Based Aerodynamic Design Optimization: Use of Surrogates in Aerodynamic Design Optimization. Int Conf Aerosp Sci Aviat Technol. 2009;13: 1–26. doi:10.21608/asat.2009.23442

[48] Voit EO. Modelling metabolic networks using power-laws and S-systems. Essays Biochem. 2008;45: 29–40. 18793121 10.1042/BSE0450029

[49] Voit EO. Biochemical Systems Theory: A Review. Raffelsberger W, Pérez-Correa R, editors. ISRN Biomath. 2013;2013: 897658. doi:10.1155/2013/897658

[50] Grimm V, Berger U, Bastiansen F, Eliassen S, Ginot V, Giske J, A standard protocol for describing individual-based and agent-based models. Ecol Model. 2006;198: 115–126. doi:10.1016/j.ecolmodel.2006.04.023

[51] Waage P, Guldberg CM. ChemTeam: Studies Concerning Affinity. In: https://www.chemteam.info/Chem-History/Concerning-Affinity.html [Internet]. [cited 3 Dec 2021]. Available: https://www.chemteam.info/Chem-History/Concerning-Affinity.html

[52] Guldberg CM, Waage P. Etudes sur les affinités chimiques. Imprimerie de Brøgger & Christie; 1867.

[53] Waage P, Guldberg C. Studier over affiniteten. Forh Vidensk-Selsk Christiania. 1864;1: 35–45.

[54] Guldberg CM, Waage P. Uber die chemische Affinität. J Prakt Chem. 1879;127: 69–114.

[55] Voit EO, Martens HA, Omholt SW. 150 Years of the Mass Action Law. PLOS Comput Biol. 2015;11: e1004012. doi:10.1371/journal.pcbi.1004012 25569257 PMC4288704

[56] Kermack WO, McKendrick AG. A contribution to the mathematical theory of epidemics. Proc R Soc Lond A. 1927;115: 700–721. doi:10.1098/rspa.1927.0118

[57] Lotka AJ. Elements of physical biology. Williams & Wilkins; 1925.

[58] Volterra V. Variazioni e fluttuazioni del numero d’individui in specie animali conviventi. 1926.

[59] Henri V. Lois générales de l’action des diastases. Paris : Librairie Scientifique A. Hermann; 1903. Available: http://archive.org/details/b28114024

[60] Michaelis L, Menten M. Die Kinetic der Invertinwirkung, 1913. Biochem Z. 49: 333.

[61] Briggs GE, Haldane JBS. A note on the kinetics of enzyme action. Biochem J. 1925;19: 338. 16743508 10.1042/bj0190338 PMC1259181

[62] Monod J. The growth of bacterial cultures. Annu Rev Microbiol. 1949;3: 371–394.

[63] Olivença DV, Davis JD, Voit EO. Comparison Between Lotka-Volterra and Multivariate Autoregressive Models of Ecological Interaction Systems. 2021 Oct p. 2021.10.07.463461. doi:10.1101/2021.10.07.463461 PMC9815445 36619477

[64] Voit EO, Davis JD, Olivença DV. Inference and Validation of the Structure of Lotka-Volterra Models. 2021 Aug p. 2021.08.14.456346. doi:10.1101/2021.08.14.456346

[65] Stein RR, Bucci V, Toussaint NC, Buffie CG, Rätsch G, Pamer EG, Ecological Modeling from Time-Series Inference: Insight into Dynamics and Stability of Intestinal Microbiota. PLOS Comput Biol. 2013;9: e1003388. doi:10.1371/journal.pcbi.1003388 24348232 PMC3861043

[66] Goel G, Chou I-C, Voit EO. System estimation from metabolic time-series data. Bioinformatics. 2008;24: 2505–2511. doi:10.1093/bioinformatics/btn470 18772153 PMC2732280

[67] Voit E, Chou I-C. Parameter estimation in canonical biological systems models. Int J Syst Synth Biol. 2010;1: 1–19.

[68] Savageau MA. Biochemical systems analysis: I. Some mathematical properties of the rate law for the component enzymatic reactions. J Theor Biol. 1969;25: 365–369. doi:10.1016/S0022-5193(69)80026-3 5387046

[69] Savageau MA. Biochemical systems analysis: II. The steady-state solutions for an n-pool system using a power-law approximation. J Theor Biol. 1969;25: 370–379. doi:10.1016/S0022-5193(69)80027-5 5387047

[70] Savageau MA. Biochemical systems analysis. 3. Dynamic solutions using a power-law approximation. J Theor Biol. 1970;26: 215–226. doi:10.1016/s0022-5193(70)80013-3 5434343

[71] Voit EO, Savageau MA. Power-law approach to modeling biological systems. II. Application to ethanol production. J Ferment TechnolJapan. 1982;60.

[72] Savageau MA, Voit EO. Power-law approach to modeling biological systems: I. Theory. J Ferment Technol. 1982;60: 221–228.

[73] Wu L, Wang W, van Winden WA, van Gulik WM, Heijnen JJ. A new framework for the estimation of control parameters in metabolic pathways using lin-log kinetics. Eur J Biochem. 2004;271: 3348–3359. doi:10.1111/j.0014-2956.2004.04269.x 15291812

[74] Visser D, Heijnen JJ. Dynamic simulation and metabolic re-design of a branched pathway using linlog kinetics. Metab Eng. 2003;5: 164–176. doi:10.1016/S1096-7176(03)00025-9 12948750

[75] Heijnen JJ. Approximative kinetic formats used in metabolic network modeling. Biotechnol Bioeng. 2005;91: 534–545. doi:10.1002/bit.20558 16003779

[76] Sasidharakurup H, Kumar G, Nair B, Diwakar S. Mathematical Modeling of Severe Acute Respiratory Syndrome Coronavirus 2 Infection Network with Cytokine Storm, Oxidative Stress, Thrombosis, Insulin Resistance, and Nitric Oxide Pathways. OMICS J Integr Biol. 2021;25: 770–781. doi:10.1089/omi.2021.0155 34807729

[77] Voit EO. Mesoscopic modeling as a starting point for computational analyses of cystic fibrosis as a systemic disease. Biochim Biophys Acta. 2014;1844: 258–270. doi:10.1016/j.bbapap.2013.03.023 23570976 PMC3770779

[78] Schulz AR. Interpretation of nutrient-response relationships in rats. J Nutr. 1991;121: 1834–1843. doi:10.1093/jn/121.11.1834 1941192

[79] Tang Y, Gupta A, Garimalla S, MaHPIC Consortium. Electronic address: http://systemsbiology.emory.edu, Galinski MR, Styczynski MP, Metabolic modeling helps interpret transcriptomic changes during malaria. Biochim Biophys Acta Mol Basis Dis. 2018;1864: 2329–2340. doi:10.1016/j.bbadis.2017.10.023 29069611 PMC5911422

[80] Vilela M, Chou I, Vinga S, Vasconcelos A, Voit E, Almeida J. Parameter optimization in S-system models. BMC Syst Biol. 2008;2. doi:10.1186/1752-0509-2-35 18416837 PMC2333970

[81] Voit E. A first course in systems biology. Garland Science; 2017.

[82] Pekalski A, Stauffer D. Three Species Lotka–Volterra Model. Int J Mod Phys C. 1998;9: 777–783.

[83] Coorey G, Figtree GA, Fletcher DF, Redfern J. The health digital twin: advancing precision cardiovascular medicine. Nat Rev Cardiol. 2021;18: 803–804. 34642446 10.1038/s41569-021-00630-4

[84] Laubenbacher R, Sluka JP, Glazier JA. Using digital twins in viral infection. Science. 2021;371: 1105–1106. doi:10.1126/science.abf3370 33707255 PMC8170388

[85] Laubenbacher R, Niarakis A, Helikar T, An G, Shapiro B, Malik-Sheriff RS, Building digital twins of the human immune system: toward a roadmap. NPJ Digit Med. 2022;5: 64. doi:10.1038/s41746-022-00610-z 35595830 PMC9122990

[86] An G, Cockrell C. Drug development digital twins for drug discovery, testing and repurposing: A schema for requirements and development. Front Syst Biol. 2022;2. doi:10.3389/fsysb.2022.928387 PMC9351294 35935475

[87] Tibshirani R. Regression Shrinkage and Selection via the Lasso. J R Stat Soc Ser B Methodol. 1996;58: 267–288.

[88] Nardini JT, Baker RE, Simpson MJ, Flores KB. Learning differential equation models from stochastic agent-based model simulations. J R Soc Interface. 2021;18: 20200987. doi:10.1098/rsif.2020.0987 33726540 PMC8086865

[89] Bottou L, Curtis FE, Nocedal J. Optimization methods for large-scale machine learning. SIAM Rev. 2018;60: 223–311.

[90] Chou I-C, Martens H, Voit EO. Parameter estimation in biochemical systems models with alternating regression. Theor Biol Med Model. 2006;3: 25. doi:10.1186/1742-4682-3-25 16854227 PMC1586003

[91] Brunton SL, Kutz JN. Data-driven science and engineering: Machine learning, dynamical systems, and control. Cambridge University Press; 2019.

[92] Schneider T, Dunbar OR, Wu J, Böttcher L, Burov D, Garbuno-Inigo A, Epidemic management and control through risk-dependent individual contact interventions. PLOS Comput Biol. 2022;18: e1010171. 35737648 10.1371/journal.pcbi.1010171 PMC9223336

[93] Böttcher L, Asikis T, Fragkos I. Control of Dual-Sourcing Inventory Systems Using Recurrent Neural Networks. Inf J Comput. 2023.

[94] Asikis T, Böttcher L, Antulov-Fantulin N. Neural ordinary differential equation control of dynamics on graphs. Phys Rev Res. 2022;4: 013221.

[95] Böttcher L, Asikis T. Near-optimal control of dynamical systems with neural ordinary differential equations. Mach Learn Sci Technol. 2022;3: 045004.

[96] Böttcher L, Antulov-Fantulin N, Asikis T. AI Pontryagin or how artificial neural networks learn to control dynamical systems. Nat Commun. 2022;13: 333. 35039488 10.1038/s41467-021-27590-0 PMC8763915

[97] Böttcher L, Fonseca LL, Laubenbacher RC. Control of Medical Digital Twins with Artificial Neural Networks. arXiv; 2024. doi:10.48550/arXiv.2403.13851 PMC11904622 40078154

[98] Wagener N, Cheng C-A, Sacks J, Boots B. An Online Learning Approach to Model Predictive Control. In: Bicchi A, Kress-Gazit H, Hutchinson S, editors. Robotics: Science and Systems XV, University of Freiburg, Freiburg im Breisgau, Germany, June 22–26, 2019. 2019. doi:10.15607/RSS.2019.XV.033

