# Supplementary material for: Surrogate modeling and control of medical digital twins
Source: ArXiv. 2024 May 20:arXiv:2402.05750v2. Preprint. [Version 2] (PMC11142319)
Supplement: Supplement 1 [file NIHPP2402.05750v2-supplement-1.pdf]

Supplementary Text 1

The sheep-wolves-grass model

The process of approximating an ODE to an ABM will be illustrated using the sheep-wolves-grass version of the Wolf Sheep Predation model[1] supplied with NetLogo[2]. The model was used as in the NetLogo database, with the exception that the size of the world was increased 25-fold, from a size of 51×51 to 255×255. Similarly, the initial populations were also increased 25-fold (sheep were increased from 100 to 2,500, and wolves from 50 to 1250), thus maintaining the initial density of agents in the world (See file 'Wolf Sheep Predation bigworld.nlogo'). Every patch in the world has a 50/50 probability of being available as green grass at the start of a simulation per definition of the original model, and this parameter was not changed. This bigger world made the simulations less noisy and allowed them to be more robust against extinction. Averaging was done by accumulating 100 runs of the same instantiation, which resulted in a similar amount of noise as with 2,500 simulations of the original 51×51 model. Under these conditions, this model has a stable steady state that is reached in approximately 300 time-steps, after which the total grass will remain around 24,290, total number of sheep around 4,001, and total number of wolves around 1,917.

To examine all ODE surrogate models, including those designed for a steady state and those not requiring steady state conditions, we formulated a control problem that targets a steady state. The control problem used is the following: We want to determine the number of wolves and sheep that need to be removed in order to move the steady state to a new point where there are only 50% of the wolves present and sheep go up to 110% relative to the original steady state. Additionally, we want the solution that results in the removal of the smallest number of animals. This results in a classical control problem with input matrix $B=diag(0,1,1)$, and control input $u=[0,-\kappa_2 Y,-\kappa_3 Z]^T$, where X is the total amount of grass, Y the total number of sheep and Z the total number of wolves.

Dataset I (Fig S1, w/o control; dataset created by simulation of the model in the file 'Wolf Sheep Predation bigworld.nlogo') was generated using 1,250 initial wolves, 2,500 initial sheep, and the original distribution of grass. Dataset II differs from dataset I by having initially more sheep and wolves but less grass. Dataset II (dataset created by simulation of the model in the file 'Wolf Sheep Predation bigworld 2nd DataSet.nlogo') was generated by shifting the original model parameters to different values (sheep energy gain from food from 4 to 5, sheep reproduction rate from 4% to 5% per timestep, and wolf reproduction rate from 5% to 1% per timestep). After 1,000 timesteps when the system reached a new steady state, the parameters were changed back to the original values (4, 4, and 5, respectively) and dataset II recorded until the system settled in its original steady state (i.e., the same as in dataset I). In dataset II, all three species exhibit wider fluctuations than in dataset I (Fig S1).

The datasets with control (III-V, Fig S1) were generated using the same initial condition and parameters as for dataset I. The simulations were conducted over 1,000 timesteps to ensure that the ABM reached a steady state. We then exerted constant permanent control on each of the species. In dataset III (dataset created by simulation of the model in the file 'Wolf Sheep Predation bigworld_ConGrass2.nlogo'), grass control involved removing 2% of the available

grass per timestep. For dataset IV (dataset created by simulation of the model in the file 'Wolf Sheep Predation bigworld_ConSheep2.nlogo'), sheep control was associated with a 2% removal of sheep per timestep. In the case of wolves (dataset V), control involved a 1.5% removal per time-step, as a 2% removal led to their extinction (dataset created by simulation of the model in the file 'Wolf Sheep Predation bigworld_ConWolves1.5.nlogo'). Subsequently, we ran the simulations for datasets III, IV, and V for an additional 500 timesteps while applying the described controls. This ultimately led to the dynamics reaching new steady states (Fig S1).

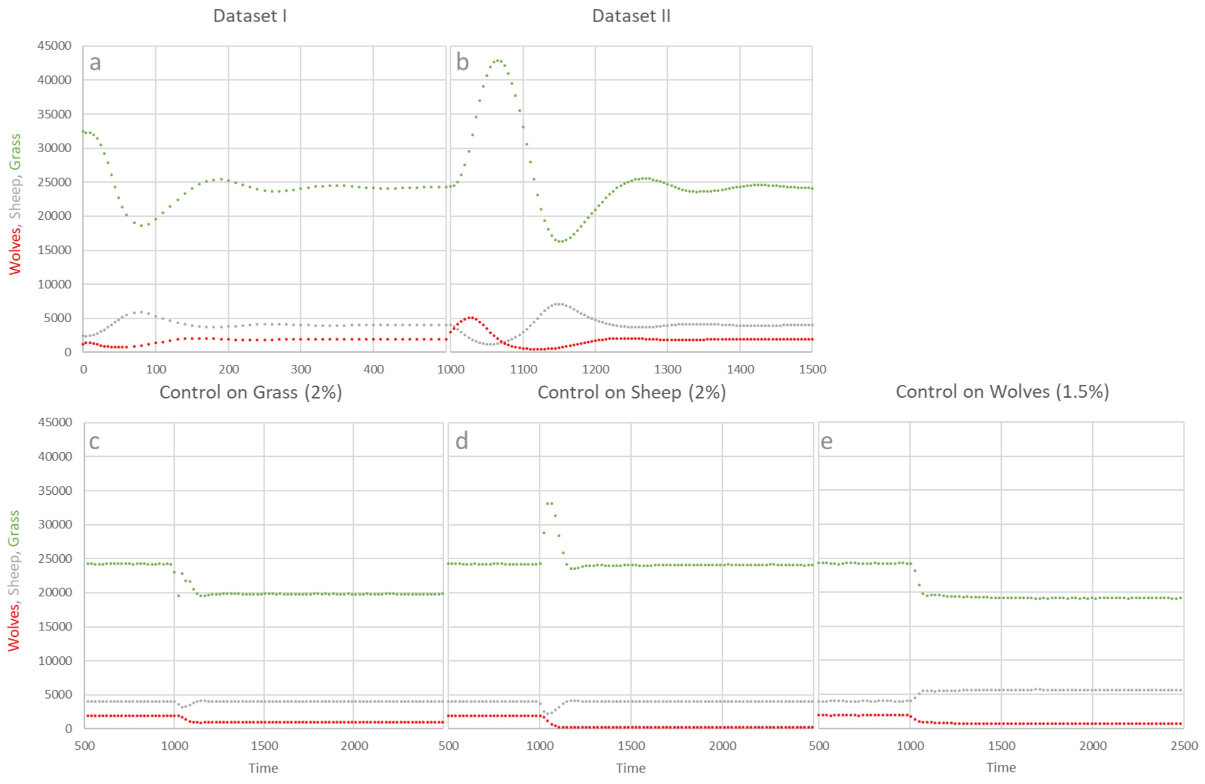

Fig S1. Training datasets for all surrogate models of the sheep-wolves-grass ABM. Datasets I and II (panels a and b) were generated with two different initial conditions. Panels c, d, and e were generated starting from the same initial conditions as dataset I (panel a) simulating until timestep 1,000, and then either 2% of grass was removed, 2% of sheep, or 1.5% of wolves.

Case 1 – Mechanistic approximation

Steps 1, 2, and 3: Analysis of the ABM shows that each patch of grass takes 30 timesteps to regrow after being consumed and the world has a maximum size, carrying capacity for grass, of 65,025. Sheep gain energy from grass consumption at a fixed rate, have a fixed probability of breeding with energy being equally divided between offspring and parent, and lose energy at a fixed rate which results in death when energy is depleted. Wolves gain energy from sheep consumption at a fixed rate, have a fixed probability of breeding with energy being equally divided between offspring and parent, and lose energy at a fixed rate which results in death when energy is depleted. All interaction processes depend only on the co-localization of the two agents

and are independent of all agent attributes including position. However, sheep and wolves do have a death process that is dependent on their energy, which could require sheep's and wolves' energies to be represented by state variables (Fig S2). Because energy acts as an inhibitor for the death of sheep and wolves, and inhibitory processes are not well-represented by mass action kinetics, we simplified the model by reassigning the negative impact of food (grass for sheep and sheep for wolves) on their mortality to a positive effect on their respective growth. This adjustment allows us to not include energy terms for both sheep and wolves in our ODE model (Fig S2). The resulting ODE model is

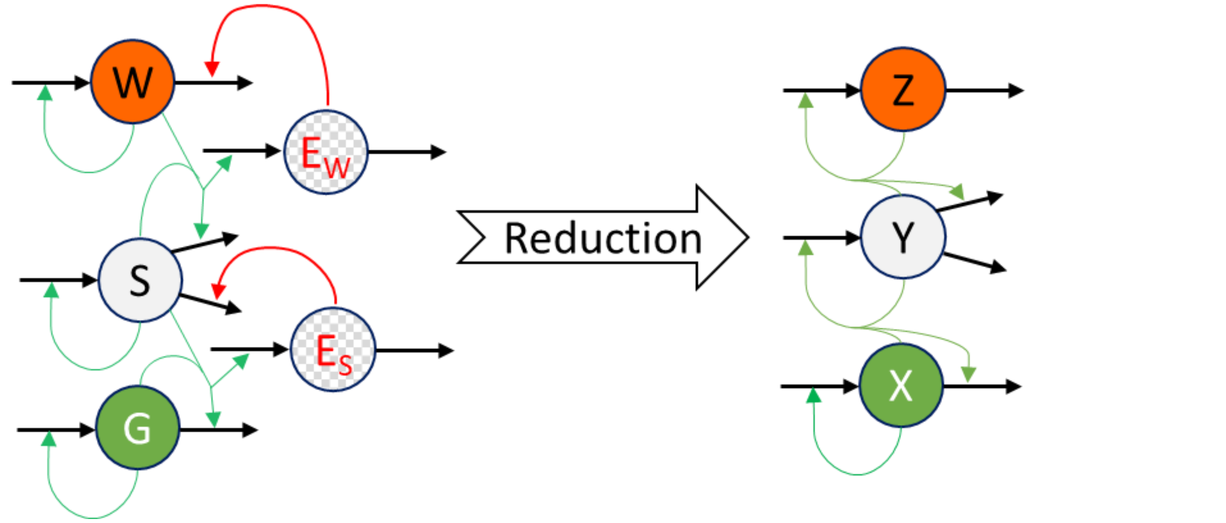

Fig S2. Model reduction from the sheep-wolves-grass ABM to the ODE surrogate model. In the ABM, the energy of sheep (E_S) and wolves (E_W) agents prevents (inhibits) their death. When the energy reaches zero, the agent dies. Generation of offspring is an event that occurs at every timestep and depends only of the probability of reproduction. After reproduction, energy is divided between parent and offspring. In order to approximate a mechanistic mass action ODE model, the inhibitory effect of energy of the agent on its death was reassigned to a positive effect on the growth of the population. W, S, and G denotes wolves, sheep and grass agents in the ABM. Z, Y, and X denote wolves, sheep and grass populations in the ODE model.

$$\frac{d}{dt}\begin{bmatrix} X \\ Y \\ Z \end{bmatrix} = M \cdot F, \qquad M = \begin{bmatrix} 1 & -1 & 0 & 0 & 0 & 0 & 0 \\ 0 & 0 & 1 & -1 & -1 & 0 & 0 \\ 0 & 0 & 0 & 0 & 0 & 1 & -1 \end{bmatrix},$$

$$F = \begin{bmatrix} k_1 \cdot X - k_2 \cdot X^2 \\ k_3 \cdot X \cdot Y \\ k_4 \cdot X \cdot Y \\ k_5 \cdot Y \\ k_6 \cdot Y \cdot Z \\ k_7 \cdot Y \cdot Z \\ k_8 \cdot Z \end{bmatrix}, \qquad \text{(Eq. 1.1)}$$

or simply

$$\dot{X} = k_1 \cdot X - k_2 \cdot X^2 - k_3 \cdot X \cdot Y$$
$$\dot{Y} = k_4 \cdot X \cdot Y - k_5 \cdot Y - k_6 \cdot Y \cdot Z, \quad \text{(Eq. 1.2)}$$
$$\dot{Z} = k_7 \cdot Y \cdot Z - k_8 \cdot Z$$

where X, Y, and Z are the total amounts of grass, sheep, and wolves, respectively, in the world, and k_i are the rate constants of the different processes.

Step 4: As described above, the control of the ABM is done by removal of a fixed percentage of the agents per timestep. This is easily approximated in an ODE by linear terms (Eq. 2).

$$\dot{X} = k_1 \cdot X - k_2 \cdot X^2 - k_3 \cdot X \cdot Y$$
$$\dot{Y} = k_4 \cdot X \cdot Y - k_5 \cdot Y - k_6 \cdot Y \cdot Z - \kappa_2 \cdot Y \quad \text{(Eq. 2)}$$
$$\dot{Z} = k_7 \cdot Y \cdot Z - k_8 \cdot Z - \kappa_3 \cdot Z$$

Step 5: To compare the effect of using datasets obtained with and without control, two parameterizations were obtained. The first parameterization was done against datasets I and II (Fig S3, (see file 'SWG_Case1_Mech.I_II.m')), and the second parameterization against all five datasets I-V (Fig S4, see file 'SWG_Case1_Mech.I_V.m'). Both parameterizations were done using least-square non-linear regression. An initial guess of the parameters was obtained using the time course slope method[3,4]. Alternatively, other methods specific for parameter optimization of Lotka–Volterra models may also be used[5]. The ODE models obtained are in good agreement with the corresponding datasets (Figs S3 and S4).

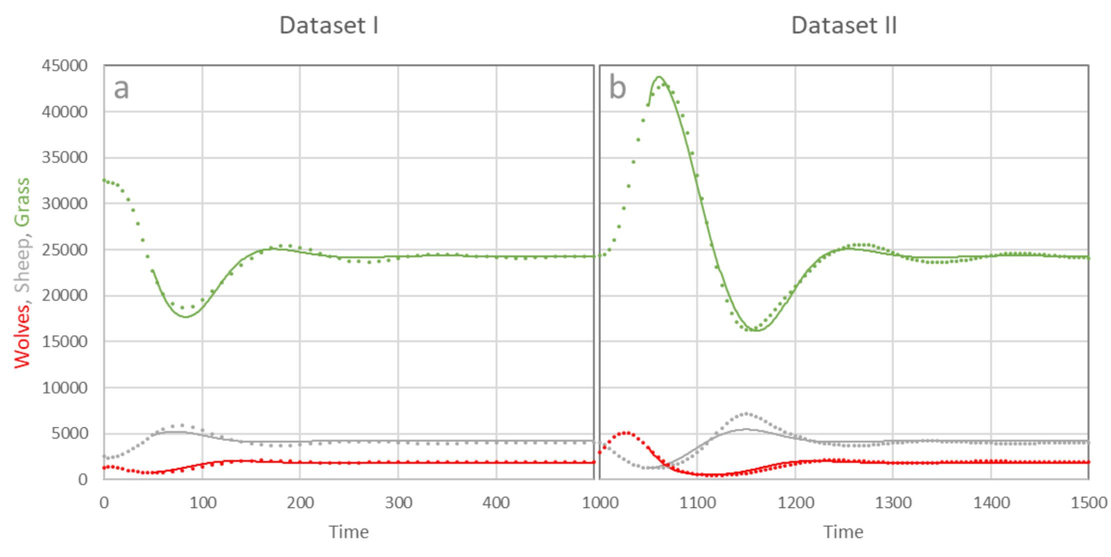

Fig S3. Fit of the mechanistic approximation (Case 1) to the ABM datasets I&II. The ODE model is shown as solid lines and training data as colored markers.

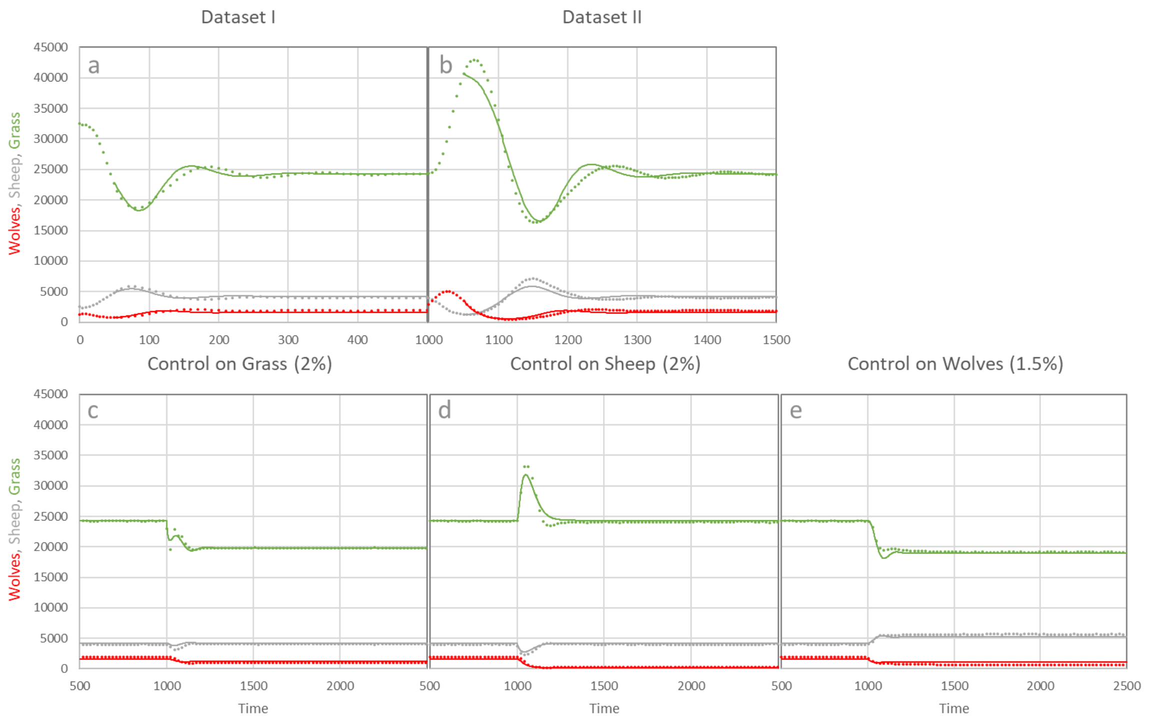

Fig S4. Fit of the mechanistic approximation (Case 1) to the ABM datasets I-V. The ODE model is shown as solid lines and training data as colored markers.

Step 6: Solving the control problem. Using the model above (Eq. 2) and the two parameterizations, κ_2 and κ_3 were determined so that this model (Eq. 2) has a steady state with 50% of the wolves and 110% of the sheep relative to the original model (Eq. 1.2). The results (mechanistic (I and II) and mechanistic (I-V)) are plotted in comparison with the best solutions found for the ABM, determined by performing a grid search on κ_2 and κ_3 (Fig S5).

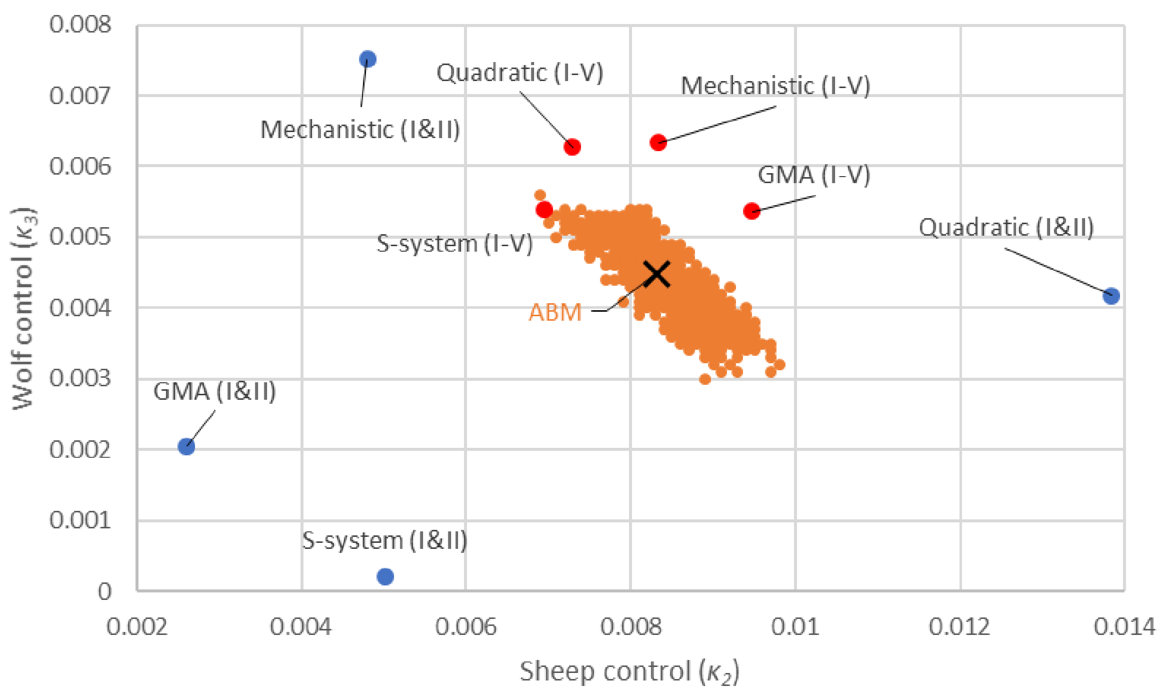

Fig S5. Comparison of effectiveness of different ODE surrogate models for solving the sheep-wolves-grass ABM control problem. The black cross marks the optimal solution (κ_2 = 0.83% and κ_3 = 0.45% per timestep) for the sheep-wolves-grass ABM control problem as determined by a grid search (with a step of 0.0001 in both dimensions). Orange dots indicate suboptimal control solutions within one standard deviation from the target (a steady state with 50% fewer wolves and 10% more sheep compared to the original steady state). Blue and red dots show the control parameter values associated with the ODE **surrogates** that have been calibrated against datasets I and II and datasets I-V, respectively. The best solutions were obtained for **surrogates** parameterized with datasets containing control information (III-V). However, all four ODE **surrogates** identified control solutions (red dots) equally distant from the optimal one.

Case 2 – GMA approximation

Depending on the complexity of the ABM being approximated, it might not be possible or desirable to perform mechanistic approximations to each and every process. One way to avoid having to assume or deduce mechanistic formulations is to use a canonical approach. In biochemical systems theory (BST) *(61,62,73)*, all processes are canonically represented by power laws

$$F_i = \alpha_i \prod_{j=1}^{m} X_j^{g_{ij}}. \qquad \text{(Eq. 3)}$$

In BST, if each process of a system is approximated as a power law this is referred to as a generalized mass action (GMA) model. On the other hand, if all processes into each state variable are approximated to a power law and all processes out of each state variable are approximated to another power law then this is referred to as an S-system model.

Step 3: The difference relative to case 1 will be that here the processes are represented by power laws of all three state variables. Thus, no assumptions are being made on which variables each of the processes depends on, although this could be done and would result in fewer parameters. Rather this dependence will be inferred from the datasets by optimization. The GMA ODE is given by

$$\frac{d}{dt}\begin{bmatrix} X \\ Y \\ Z \end{bmatrix} = M \cdot F, \qquad M = \begin{bmatrix} 1 & -1 & 0 & 0 & 0 & 0 & 0 \\ 0 & 0 & 1 & -1 & -1 & 0 & 0 \\ 0 & 0 & 0 & 0 & 0 & 1 & -1 \end{bmatrix}, \qquad \text{(Eq. 4)}$$
$$F_i = \alpha_i \cdot X^{g_{i1}} \cdot Y^{g_{i2}} \cdot Z^{g_{i3}}, \qquad i \in \{1,2,3,4,5,6,7\},$$

which shares the same stoichiometric matrix (M) with case 1 (Eq. 1.2).

Step 4: Control terms were approximated as in case 1, which yields a similar model

$$\frac{d}{dt}\begin{bmatrix} X \\ Y \\ Z \end{bmatrix} = M \cdot F - \begin{bmatrix} 0 \\ \kappa_2 \cdot Y \\ \kappa_3 \cdot Z \end{bmatrix},$$
$$M = \begin{bmatrix} 1 & -1 & 0 & 0 & 0 & 0 & 0 \\ 0 & 0 & 1 & -1 & -1 & 0 & 0 \\ 0 & 0 & 0 & 0 & 0 & 1 & -1 \end{bmatrix}, \quad F_i = \alpha_i \cdot X^{g_{i1}} \cdot Y^{g_{i2}} \cdot Z^{g_{i3}}, \qquad \text{(Eq. 5)}$$

where $i \in \{1, 2, 3, 4, 5, 6, 7\}$.

Step 5: As in the mechanistic approximation (case 1), we optimized the model against two sets of datasets for comparison. In the first (Case 2.1, GMA (I and II)), since not all variables are expected to regulate each of the processes, an L1-regularization term was added to the objective function that leads to the selection of the best fitted model with the fewest number of kinetic orders different from zero. This L1 approach is similar to what is used in the LASSO method[6]. We performed this optimization using only datasets I and II. In the second approach (Case 2.2, GMA (I-V)), we took advantage of all parameters and performed the optimization (regular non-linear optimization) against all five datasets (I-V). Datasets III-V were used by exerting control on each of the variables by setting *(κ_1, κ_2, κ_3)* to either [0.02, 0, 0], [0, 0.02, 0], or [0, 0, 0.015], respectively, in equation Eq. 6.

$$\frac{d}{dt}\begin{bmatrix} X \\ Y \\ Z \end{bmatrix} = M \cdot F - \begin{bmatrix} \kappa_1 \cdot X \\ \kappa_2 \cdot Y \\ \kappa_3 \cdot Z \end{bmatrix},$$
$$M = \begin{bmatrix} 1 & -1 & 0 & 0 & 0 & 0 & 0 \\ 0 & 0 & 1 & -1 & -1 & 0 & 0 \\ 0 & 0 & 0 & 0 & 0 & 1 & -1 \end{bmatrix}, \quad F_i = \alpha_i \cdot X^{g_{i1}} \cdot Y^{g_{i2}} \cdot Z^{g_{i3}}, \qquad \text{(Eq. 6)}$$

where $i \in \{1, 2, 3, 4, 5, 6, 7\}$.

The first GMA model (Case 2.1, see file 'SWG_Case2_GMA.I_II.m') fitted well both datasets I and II (Fig S6). Interestingly, three of the kinetic orders were not needed for model fit, and so in this model, grass does not regulate F_5, sheep removal by wolves, and F_7, death of wolves. This latter process is also not dependent on sheep (Table S1). This effectively lowered the number of parameters to 25. Three kinetic orders were found to be negative: (i) grass' effect on grass growth, (ii) grass' effect on death of sheep, and (iii) wolves' effect on grass consumption by sheep (Table S1). These are interesting results as grass does technically inhibit the death of sheep, since in the ABM lack of energy causes sheep to die, and results confirm that the ABM

simulations contained evidences of grass inhibiting the death of sheep. The inhibition of sheep consumption of grass by wolves (g_{23} in F_2) makes sense only as an indirect effect, since wolves do not play an explicit role in that process, but higher levels of wolves do lead to decreased levels of sheep, which then eat less grass. Interestingly, the process of wolf removal was not found to be inhibited by sheep, which was expected given that in the considered ABM wolves die only due to a lack of energy.

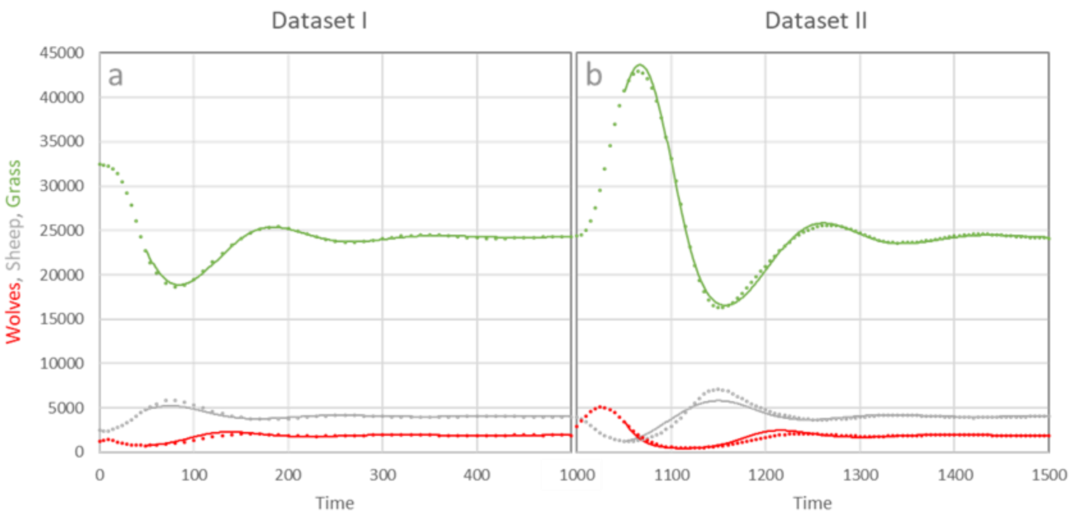

Fig S6. Fit of the GMA approximation (Case 2) to the ABM datasets I&II. GMA approximation (solid lines) and corresponding training data (colored markers).

Table S1. Parameters obtained for the processes of the canonical ODE model. All values were rounded to two decimal places.

Processes (i)	Rate constants (i)	Kinetic Orders		
		g_{i1}	g_{i2}	g_{i3}
P_1	$7.08 \cdot 10^2$	-0.13	0.07	0.14
P_2	$2.74 \cdot 10^{-5}$	1.01	0.92	-0.06
P_3	$1.18 \cdot 10^{-6}$	0.94	0.99	0.06
P_4	7.64	-0.94	0.68	0.70
P_5	$2.48 \cdot 10^{-6}$	0	1.44	0.66
P_6	$1.09 \cdot 10^{-5}$	0.04	1.00	1.04
P_7	$1.29 \cdot 10^{-1}$	0	0	0.94

A second model was created by fitting the GMA model (Eq. 5) against all five datasets (case 2.2, GMA (I-V), see file 'SWG_Case2_GMA.I_V.m'). The model did fit well datasets III-V, yet this came at the expense of a loss of fitness towards dataset II (Fig S7).

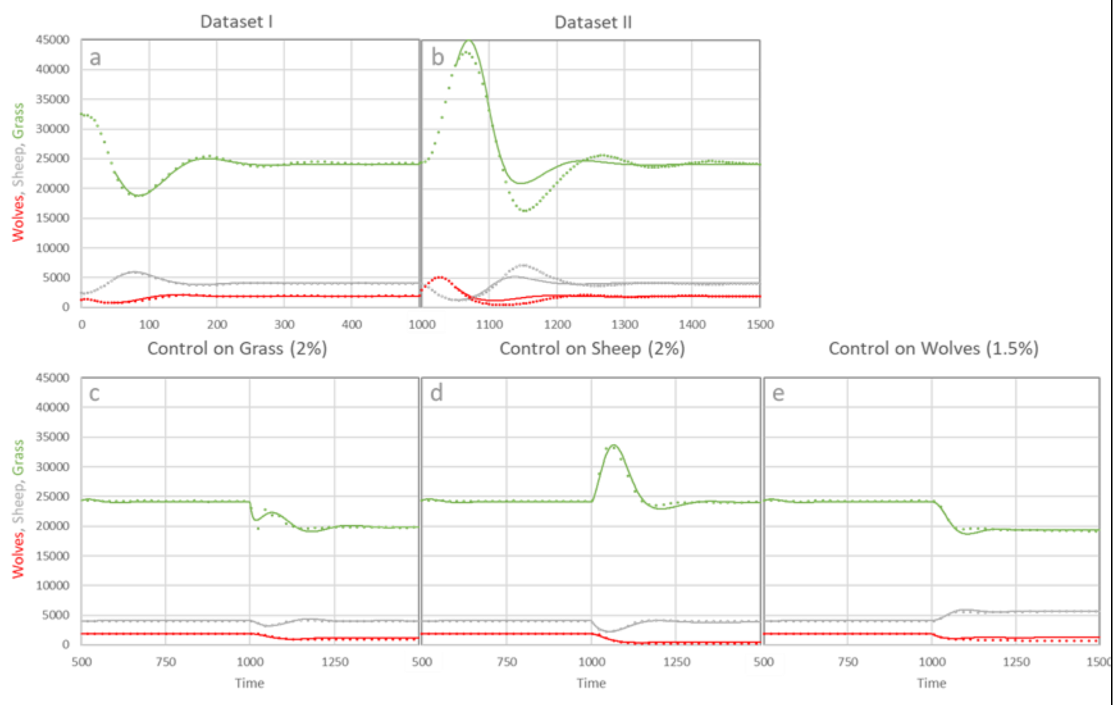

Fig S7. Fit of the GMA approximation (Case 2) to the ABM datasets I-V. GMA approximation (solid lines) and corresponding training data (colored markers).

Step 6: Both models were used to estimate the levels of control (in κ_2 and κ_3) that need to be exerted in order to reduce the steady state of wolves to 50% and increase sheep to 110% (Fig S7). Clearly the model parameterized against all 5 datasets (GMA (I-V)) performed better, predicting a control solution much closer to the ABM solution than when parameterized only against datasets I and II (GMA (I-V)).

Case 3 Approximations in the vicinity of the steady state

Generation of dynamic approximations are more complex and require the inference of the mechanisms involved in the ABM. However, when an ABM has a stable steady state of interest, and the control problem will not take the model too far away from this steady state, then it is possible to generate surrogate models anchored at the steady state. We study steady-state stability in ODEs by linear approximation around the steady state. In this context, we propose applying a similar approach to a given ABM. The difference is that the elements of the Jacobian matrix are inferred by optimization rather than analytically calculated from the derivatives of the ODEs.

Step 1: Identification of the state variables. The state variables have been defined in earlier cases. For consistency in the comparison, the same grass, sheep, and wolves will be used. We created two approximations: a linear approximation

$$\frac{d}{dt}\begin{bmatrix} X \\ Y \\ Z \end{bmatrix} = J \cdot \left(\begin{bmatrix} X \\ Y \\ Z \end{bmatrix} - \begin{bmatrix} X_{SS} \\ Y_{SS} \\ Z_{SS} \end{bmatrix} \right), \qquad \text{(Eq. 7)}$$

and a quadratic approximation

$$\frac{d}{dt}\begin{bmatrix} X \\ Y \\ Z \end{bmatrix} = J \cdot \begin{bmatrix} X - X_{SS} \\ Y - Y_{SS} \\ Z - Z_{ss} \end{bmatrix} + H \cdot \begin{bmatrix} (X - X_{SS})^2 \\ (Y - Y_{SS})^2 \\ (Z - Z_{SS})^2 \\ (X - X_{SS}) \cdot (Y - Y_{SS}) \\ (X - X_{SS}) \cdot (Z - Z_{ss}) \\ (Y - Y_{SS}) \cdot (Z - Z_{ss}) \end{bmatrix}, \qquad \text{(Eq. 8)}$$

where J is a 3×3 matrix of the first-order elements, and H is a 3×6 matrix of the second-order elements, but not a true Hessian matrix.

Step 2: Determination of the steady state point. Using the datasets I and II and previous analyses, the steady state is *$(X_{SS},Y_{SS},Z_{SS})=(24{,}290, 4{,}001, 1{,}917)$.*

Step 3: The control terms were approximated as in case 1, and the two models (Eq. 7 and 8) with the control terms are

$$\frac{d}{dt}\begin{bmatrix} X \\ Y \\ Z \end{bmatrix} = J \cdot \left(\begin{bmatrix} X \\ Y \\ Z \end{bmatrix} - \begin{bmatrix} X_{SS} \\ Y_{SS} \\ Z_{ss} \end{bmatrix} \right) - \begin{bmatrix} 0 \\ \kappa_2 \cdot Y \\ \kappa_3 \cdot Z \end{bmatrix}, \text{ and} \qquad \text{(Eq. 9)}$$

$$\frac{d}{dt}\begin{bmatrix} X \\ Y \\ Z \end{bmatrix} = J \cdot \begin{bmatrix} X - X_{SS} \\ Y - Y_{SS} \\ Z - Z_{ss} \end{bmatrix} + H \cdot \begin{bmatrix} (X - X_{SS})^2 \\ (Y - Y_{SS})^2 \\ (Z - Z_{SS})^2 \\ (X - X_{SS}) \cdot (Y - Y_{SS}) \\ (X - X_{SS}) \cdot (Z - Z_{ss}) \\ (Y - Y_{SS}) \cdot (Z - Z_{ss}) \end{bmatrix} - \begin{bmatrix} 0 \\ \kappa_2 \cdot Y \\ \kappa_3 \cdot Z \end{bmatrix}. \qquad \text{(Eq. 10)}$$

Step 4: Parameterization of the models. When we attempted to fit the linear model (Eq. 7) to the datasets I and II, a good solution could not be found. We then aimed to fit it against each of the two datasets and found better fits, although the fit against dataset I was better (Fig S8, see file 'SWG_Case3_Linear.I.m'). This underscores a limitation of this approach—it may not be able to approximate values that are too far away from the steady state.

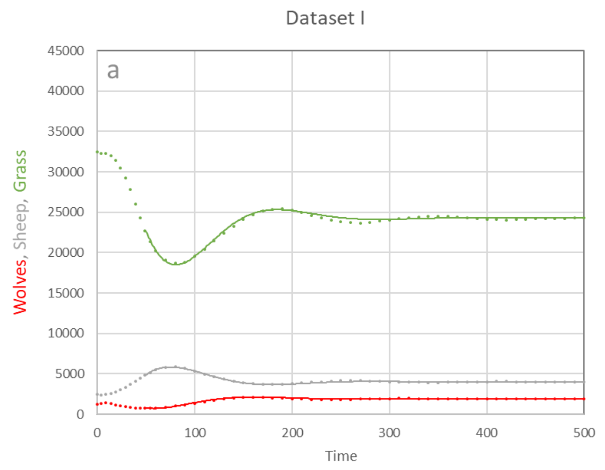

Fig S8. Fit of the Linear approximation (Case 3) to the ABM dataset I. Linear approximation (solid line) and corresponding training data (colored markers).

The quadratic model (Eq. 8), however, was able to fit both datasets I and II (see file 'SWG_Case3_Quad.I_II.m') and datasets I-V(see file 'SWG_Case3_Quad.I_V.m'). This is not surprising as this model (Eq. 8) has 27 parameters. For fitting datasets I-V, the system below was used (Eq. 11). Both optimizations generated good fits. However, when these models were used to predict the solution of the control problem, they did not perform well. The optimizations were unstable and did not converge. We recognized that second-order systems, unlike first-order systems, have more steady states and some of these are unstable. Thus, we re-parameterized the models against datasets I and II (Fig S9) and I-V (Fig S10), while excluding any parameterization that had extra steady states within the region of interest, and with this strategy it was possible to easily predict the solutions to the control problem.

$$\frac{d}{dt}\begin{bmatrix} X \\ Y \\ Z \end{bmatrix} = J \cdot \begin{bmatrix} X - X_{SS} \\ Y - Y_{SS} \\ Z - Z_{SS} \end{bmatrix} + H \cdot \begin{bmatrix} (X - X_{SS})^2 \\ (Y - Y_{SS})^2 \\ (Z - Z_{SS})^2 \\ (X - X_{SS}) \cdot (Y - Y_{SS}) \\ (X - X_{SS}) \cdot (Z - Z_{SS}) \\ (Y - Y_{SS}) \cdot (Z - Z_{SS}) \end{bmatrix} - \begin{bmatrix} \kappa_1 \cdot X \\ \kappa_2 \cdot Y \\ \kappa_3 \cdot Z \end{bmatrix} \qquad \text{(Eq. 11)}$$

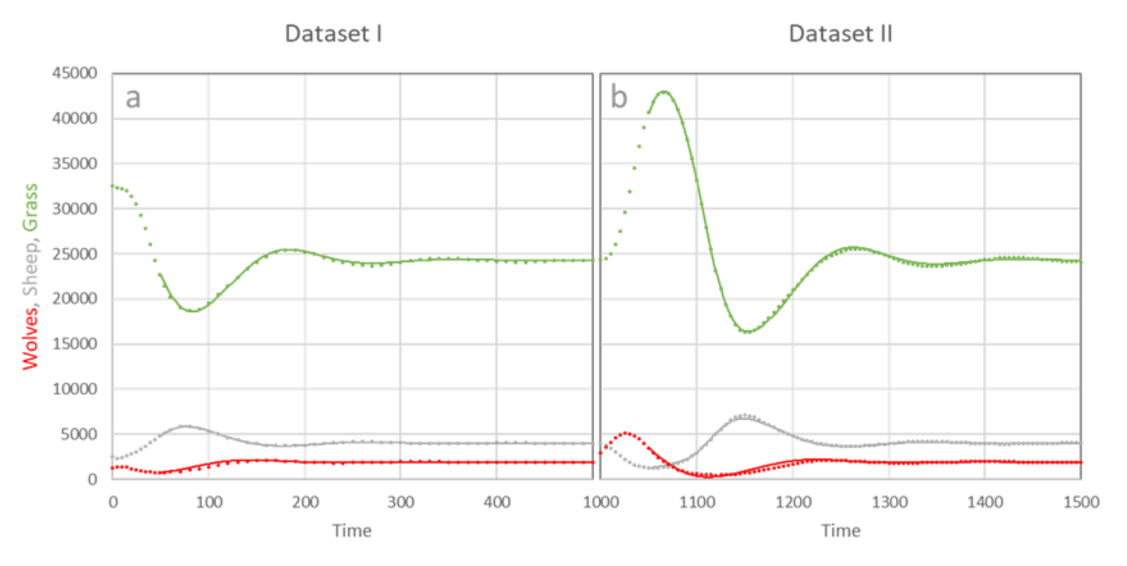

Fig S9. Fit of the Quadratic approximation (Case 3) to the ABM datasets I and II. Quadratic approximation (solid lines) and corresponding training data (colored markers).

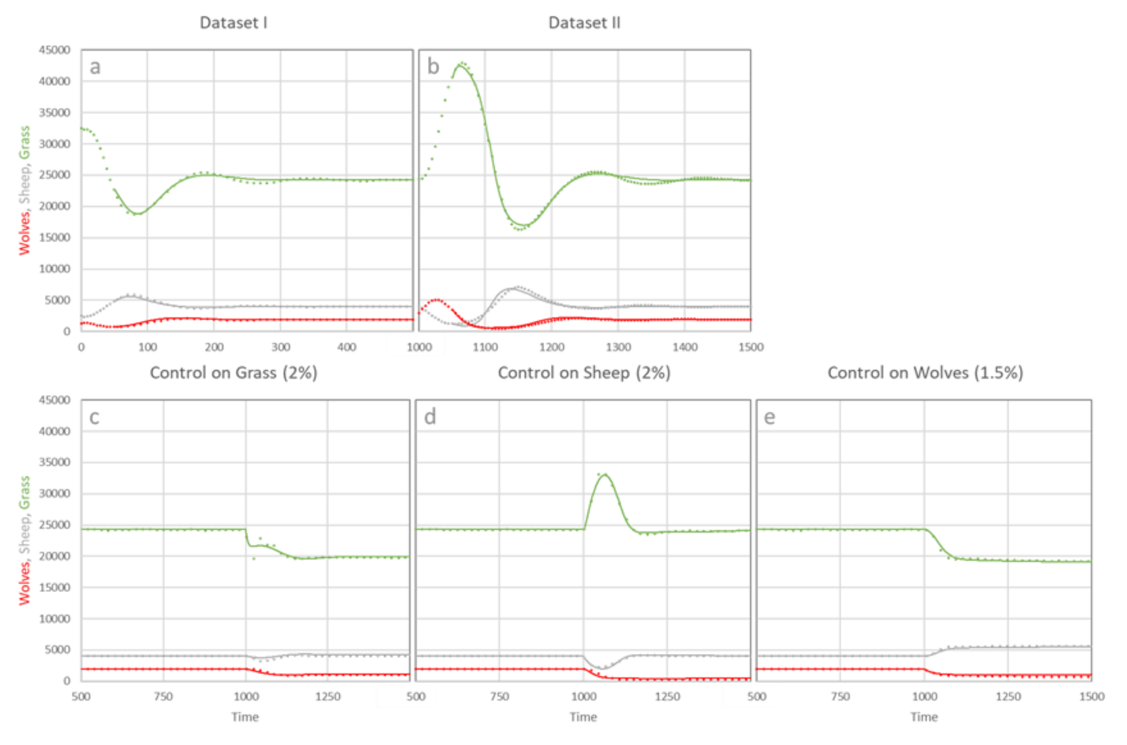

Fig S10. Fit of the Quadratic approximation (Case 3) to the ABM datasets I-V. Quadratic approximation (solid lines) and corresponding training data (colored markers).

Step 5: All three models (linear I and II, quadratic I and II, and quadratic I-V) were used to estimate the levels of control (in κ_2 and κ_3) needed to reduce the steady state of wolves to 50%

and increase sheep to 110% (Fig S5). The linear approximation predicted a control solution *(κ_2, κ_3)= (0.0884, 0.0297)* that was far from all other approaches and it is not shown in Fig S5. Both parameterizations of the quadratic approach are shown in Fig S5, and similarly to case 2 the parameterization obtained from all five datasets (I-V) predicted a much better control solution than the parameterization obtained from datasets I and II.

Case 4 The S-system approach

An S-system model allows one to approximate an ABM as a set of ODEs where each state variable is modeled as the difference between two power law terms (Eq. 12). The first term approximates all incoming processes, while the second approximates all outgoing processes.

Step 1: Identification of all state variables. We will use the same three state variables as in the previous cases: grass, sheep, and wolves. Therefore, the ODE system for a 3-variable S-system is

$$\begin{aligned}\dot{X} &= \alpha_1 \cdot X^{g_{11}} \cdot Y^{g_{12}} \cdot Z^{g_{13}} - \beta_1 \cdot X^{h_{11}} \cdot Y^{h_{12}} \cdot Z^{h_{13}} \\ \dot{Y} &= \alpha_2 \cdot X^{g_{21}} \cdot Y^{g_{22}} \cdot Z^{g_{23}} - \beta_2 \cdot X^{h_{21}} \cdot Y^{h_{22}} \cdot Z^{h_{23}}. \\ \dot{Z} &= \alpha_3 \cdot X^{g_{31}} \cdot Y^{g_{32}} \cdot Z^{g_{33}} - \beta_3 \cdot X^{h_{31}} \cdot Y^{h_{32}} \cdot Z^{h_{33}}\end{aligned} \qquad \text{(Eq. 12)}$$

Step 2: The control terms were approximated as in previous cases. The S-system model with the control terms is

$$\begin{aligned}\dot{X} &= \alpha_1 \cdot X^{g_{11}} \cdot Y^{g_{12}} \cdot Z^{g_{13}} - \beta_1 \cdot X^{h_{11}} \cdot Y^{h_{12}} \cdot Z^{h_{13}} \\ \dot{Y} &= \alpha_2 \cdot X^{g_{21}} \cdot Y^{g_{22}} \cdot Z^{g_{23}} - \beta_2 \cdot X^{h_{21}} \cdot Y^{h_{22}} \cdot Z^{h_{23}} - \kappa_2 \cdot Y. \\ \dot{Z} &= \alpha_3 \cdot X^{g_{31}} \cdot Y^{g_{32}} \cdot Z^{g_{33}} - \beta_3 \cdot X^{h_{31}} \cdot Y^{h_{32}} \cdot Z^{h_{33}} - \kappa_3 \cdot Z\end{aligned} \qquad \text{(Eq. 13)}$$

Step 3: Parameterization of the model. Similar to what was done previously, two parameterizations were generated: one against datasets I and II (Fig S11, see file 'SWG_Case4_Ssystem.I_II.m'), and a second against all five datasets I-V (Fig S12, see file 'SWG_Case4_Ssystem.I_V.m'). For the parameterization against all five datasets, we used the ODE system

$$\begin{aligned}\dot{X} &= \alpha_1 \cdot X^{g_{11}} \cdot Y^{g_{12}} \cdot Z^{g_{13}} - \beta_1 \cdot X^{h_{11}} \cdot Y^{h_{12}} \cdot Z^{h_{13}} - \kappa_1 \cdot X \\ \dot{Y} &= \alpha_2 \cdot X^{g_{21}} \cdot Y^{g_{22}} \cdot Z^{g_{23}} - \beta_2 \cdot X^{h_{21}} \cdot Y^{h_{22}} \cdot Z^{h_{23}} - \kappa_2 \cdot Y. \\ \dot{Z} &= \alpha_3 \cdot X^{g_{31}} \cdot Y^{g_{32}} \cdot Z^{g_{33}} - \beta_3 \cdot X^{h_{31}} \cdot Y^{h_{32}} \cdot Z^{h_{33}} - \kappa_3 \cdot Z\end{aligned} \qquad \text{(Eq. 14)}$$

Both optimizations produced good fits (Figs S11 and S12). Similar to cases 2 and 3, the fit was slightly less accurate when considering all five datasets compared to when considering just datasets I and II.

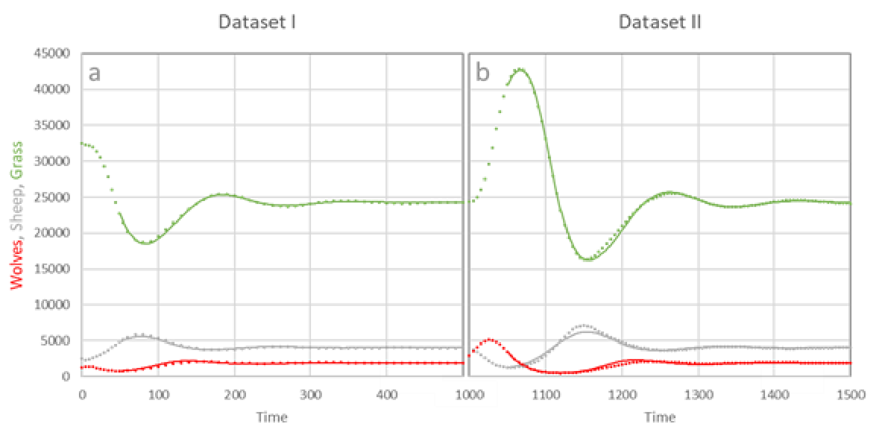

Fig S11. Fit of the S-system approximation (Case 4) to the ABM datasets I and II. The S-system approximation (solid line) and corresponding training data (colored markers).

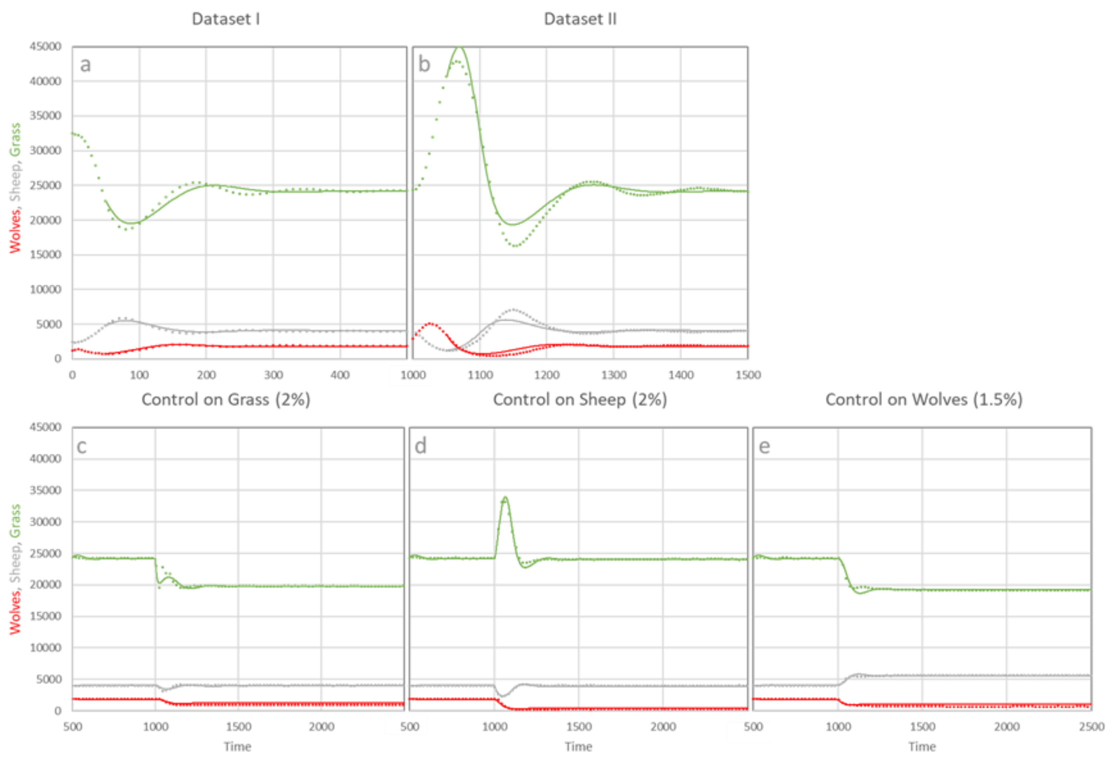

Fig S12. Fit of the S-system approximation (Case 4) to the ABM datasets I-V. The S-system approximation (solid line) and corresponding training data (colored markers).

Step 4: Both parameterizations were used to estimate the levels of control (in κ_2 and κ_3) needed to reduce the steady state of wolves to 50% and increase sheep to 110% (Fig S5). Both parameterizations of the S-system approach are shown in Fig S5, and similarly to cases 2 and 3

the parameterization obtained from all five datasets predicted a much better control solution than the parameterization obtained from datasets I and II.

The metabolic pathway model

In the first example, we considered the sheep-wolves-grass model, and trained all approximations with control data to create surrogate models. We could not identify significant differences in the ability of the considered surrogates to determine the optimal control solution. To provide further insights into the different approximations, we developed a simplified metabolic ABM. This ABM has a mechanistic approximation based on Michaelis–Menten kinetics. The use of this kinetics adds complexity, as accurately approximating Michaelis–Menten kinetics over a wide range of substrate concentrations is challenging for power laws. The pathway deliberately includes divergent processes as these are difficult to be captured accurately with S-system approximations.

The metabolic model (Fig S13) is based on four reactions catalyzed by four enzymes (A, E, I, and O), and five metabolites (S, P, Q, R, and T). Enzyme A, catalyzes the conversion of S into P, and is competitively inhibited by R, enzyme E, catalyzes the conversion of P into Q, enzyme I, catalyzes the conversion of Q into R, and enzyme O, catalyzes the conversion of P into T and is activated by R (Figs S13A and S13B). Activation of the conversion of P into T by R is modeled by setting the rate of conversion of [ORP] into [ORT] faster than the rate of conversion of [OP] into [OT] (Fig S13B). Enzyme A forms complexes AS, AP and AR, enzyme E, EP and EQ, enzyme I, IQ and IR, and enzyme O, OP, OT, OR, ORP, and ORT (Fig S13B). The model assumes all metabolites move 10 times faster than enzymes and complexes, and all species move randomly in a continuous space of 100×100 with periodic boundary conditions. A metabolite is available to bind with an enzyme or complex when they are at the same grid point, modeled by flooring (*floor* function) their positions. Two types of simulations were used, batch (Fig S13A) and continuous (Fig S13C). In batch mode, the simulations were started with 200 agents of each enzyme, 80,000 of S, 20,000 of P, 20,000 of Q, 10 of R, 10 of T, and no enzymatic complexes were present initially (Fig S14). In continuous mode, the simulations were started with the same initial conditions, but there was a constant inflow of S, at a rate of one agent per timestep and a constant removal of all metabolites, at a rate of 0.05% per timestep (Fig S15).

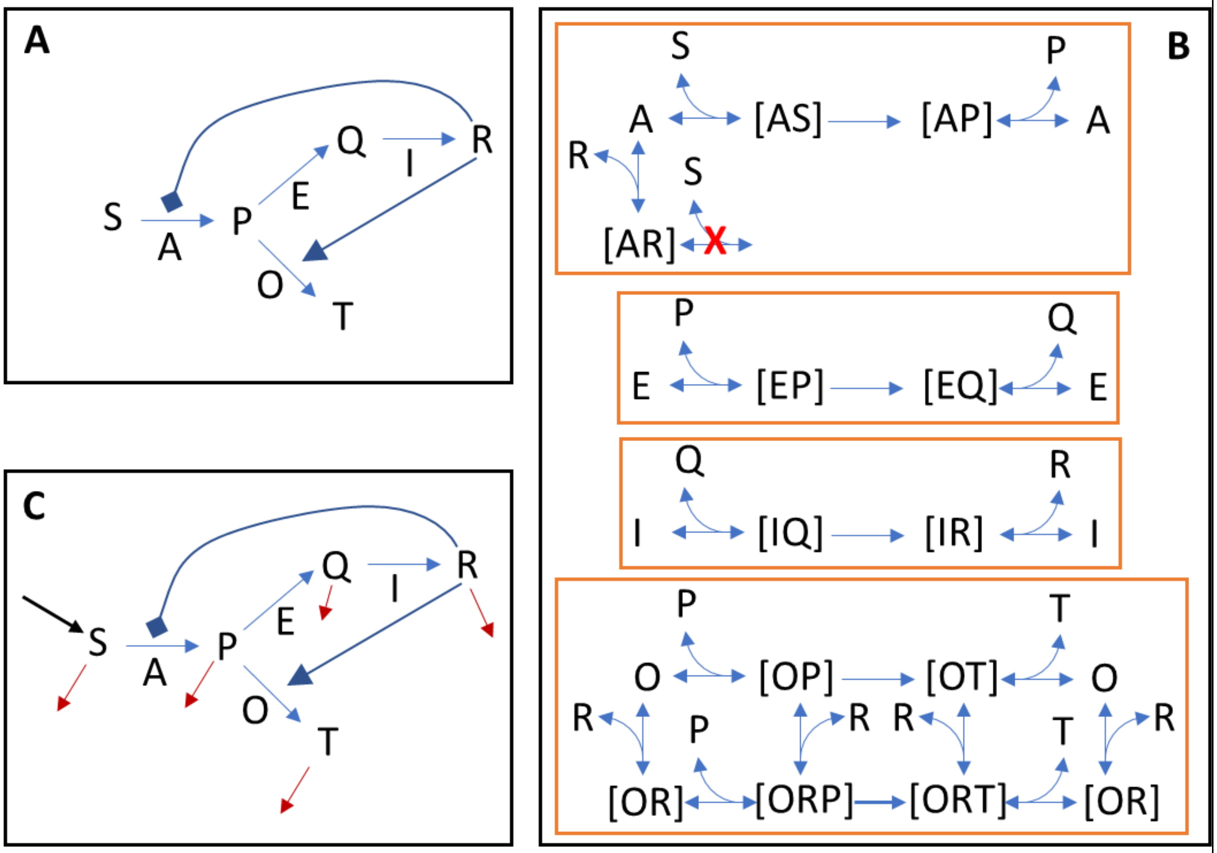

Fig S13. Metabolic toy ABM representations. (A) The diagram of the macroscopic representation of the ABM corresponding to the microscopic diagram in (B). (C) The macroscopic representation when the model is used in continuous mode, where a constant inflow of agents S occurs while all metabolites are removed at a constant rate. In (B), all pairwise interactions and complex decompositions are modeled with different probabilities. Two agents are able to interact when present in the same grid point, which was modeled with a *floor* function.

The control problem that we wish to solve is to determine the constant rate of inflow of S that minimizes the waist of S and maximizes the production of R and T, while all metabolites are removed at a rate of 0.05% per timestep. Mathematically, the goal is to identify the constant inflow, Q_{in}, that minimizes the loss function,

$$Loss(Q_{in}) = \sum_{k=1}^{N_t} \frac{S_k}{R_k + T_k}, \qquad \text{(Eq. 15)}$$

during a simulation run of N_t=50,000 timesteps, where S_k, R_k and T_k are the concentrations of S, R and T at timestep k.

To compare the ability of all proposed ODE approximations (mechanistic, GMA, S-system, quadratic, and linear), we optimized them against the two collections of datasets (‘I’ will denote models optimized against datasets I and II, generated with a single simulation of each condition; and ‘C’ will denote models optimized against datasets III-V, generated by averaging 100

simulations of each condition). Dataset I (generated with file 'Met_Pathway_dataset_80k_20k_20k_10_10_NoDil.m') was obtained as a single simulation of the metabolic ABM with the initial conditions listed above and under batch mode (Fig S14), and dataset II (generated with file 'Met_Pathway_dataset_80k_20k_20k_10_10_wDil_wFeed.m') was obtained as a single simulation of the metabolic ABM with the initial conditions listed above, under continuous mode, with an inflow rate of 1 agent of S per timestep and a removal rate of 0.05% per timestep (Fig S14). Dataset III (Fig S14) was obtained by averaging 100 simulations started in the same initial conditions as dataset I, and dataset IV (Fig S14) was obtained by averaging 100 simulations started using the same initial conditions and continuous mode as dataset II. Finally, dataset V was obtained by averaging 100 simulations started using the same initial conditions as dataset IV, and in continuous mode but with an inflow of 0.2 agents of S per timestep (Fig S14).

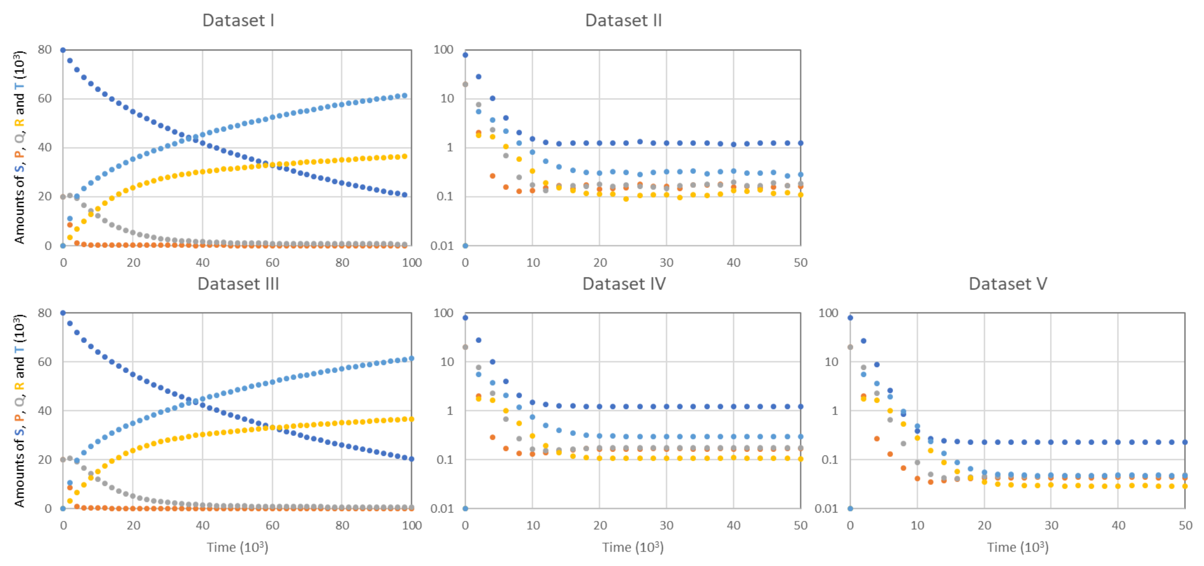

Fig S14. Datasets used to train the surrogate models for the metabolic ABM. Collection I is made of datasets I&II, and collection C is made of datasets III-V. For visualization purposes the datasets were down sampled from 1 point per timestep to 1 point per 2,000 timesteps. All calculations were performed using 1 point per timestep.

Case 1 - Mechanistic

For the mechanistic approximation each of the microscopic reactions depicted in Fig S13B were approximated to a Michaelis–Menten type rate law, which reduced the ODE model to the diagram shown in Fig S13A. The reaction catalyzed by A was approximated as a single-substrate irreversible reaction with two competitive inhibitors, P and R (Eq. 16). The reactions catalyzed by E and I were approximated as single-substrate irreversible reactions with product inhibition (Eq. 16), and the reaction catalyzed by O was approximated as a mixed noncompetitive activation (also known as heterotropic allosteric activation), where the conversion of P to T occurs faster when R is bound to O (Eq. 16). The ODE approximation is given by

$$\frac{dX}{dt} = \begin{bmatrix} -1 & 0 & 0 & 0 \\ 1 & -1 & 0 & -1 \\ 0 & 1 & -1 & 0 \\ 0 & 0 & 1 & 0 \\ 0 & 0 & 0 & 1 \end{bmatrix} \cdot \begin{bmatrix} F_A \\ F_E \\ F_I \\ F_O \end{bmatrix} + \begin{bmatrix} Q_{in} \\ 0 \\ 0 \\ 0 \\ 0 \end{bmatrix} - k_{out} \cdot X,$$

$$F_A = \frac{p_1 \cdot \frac{X_1}{p_2}}{1 + \frac{X_1}{p_2} + \frac{X_2}{p_3} + \frac{X_4}{p_4}}, F_E = \frac{p_5 \cdot \frac{X_2}{p_6}}{1 + \frac{X_2}{p_6} + \frac{X_3}{p_7}}, F_I = \frac{p_8 \cdot \frac{X_3}{p_9}}{1 + \frac{X_3}{p_9} + \frac{X_4}{p_{10}}}, \quad \text{(Eq. 16)}$$

$$F_O = \frac{p_{11} \cdot \frac{X_2}{p_{12}} + p_{13} \cdot \frac{X_4}{p_{14}} \cdot \frac{X_2}{p_{15}}}{1 + \frac{X_2}{p_{12}} + \frac{X_5}{p_{16}} + \frac{X_4}{p_{14}} \cdot \left(1 + \frac{X_2}{p_{15}} + \frac{X_5}{p_{17}}\right)},$$

where $X \in \mathbb{R}^5$ is the vector of the state variables representing the concentrations of S, P, Q, R and T, respectively; F_A, F_E, F_I, and F_O are the fluxes through the reactions catalyzed by A, E, I, and O, respectively; Q_{in} is the inflow of S when the system is in continuous mode; k_{out} is the rate of removal of metabolites when the system is in continuous mode; and $p_j \in \mathbb{R}^{17}$ is the vector of kinetic parameters.

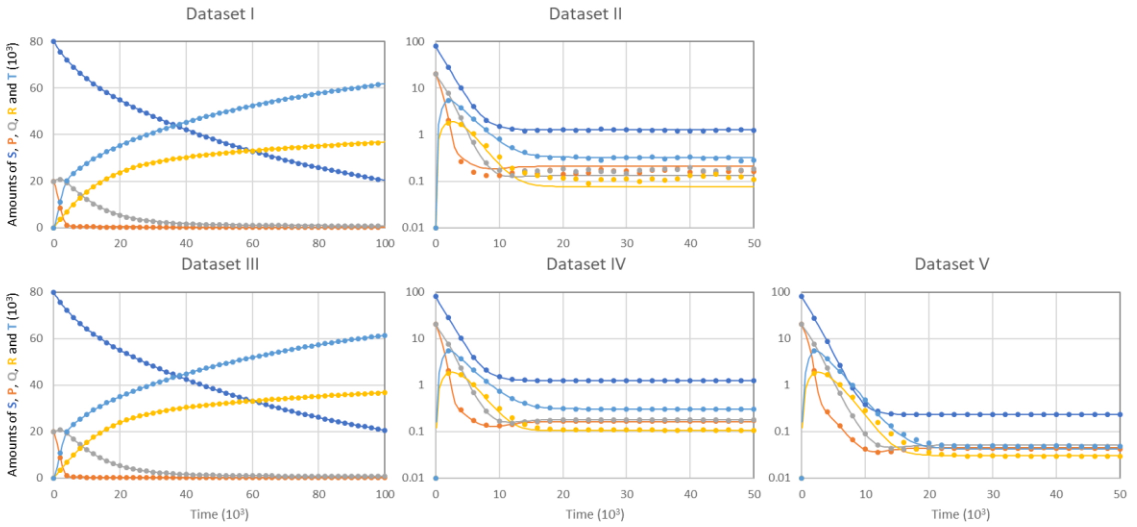

Fig S15. Fit of the mechanistic approximation (Case 1) to the ABM dataset collections I and C. The mechanistic approximation (solid line) and corresponding training data (colored markers). The Mech I model fit was obtained with datasets I&II (collection I) and the Mech C model fit was obtained with datasets III-V (collection C). For visualization purposes the datasets were down sampled from 1 point per timestep to 1 point per 2,000 timesteps.

This mechanistic ODE model was parameterized against the two collections of datasets (Fig S15), which resulted in two models: Mech. I(see file 'MetPw_Case1_Mech.I.m') and Mech. C(see file 'MetPw_Case1_Mech.C.m'). These two models were then evaluated for their ability to identify the solution to the control problem (Eq. 15), and the results are shown in Fig S16. The

solution of the control problem was also directly evaluated on the ABM by grid search in the domain of Q_{in} between 0 and 1 with a step size on 0.1. At each evaluation point, 100 simulations were averaged (Fig S16). The optimal value found for the ABM was Q_{in}=0.7. Both parameterizations of the mechanistic model were good at predicting the optimal loss function value and optimal inflow of S.

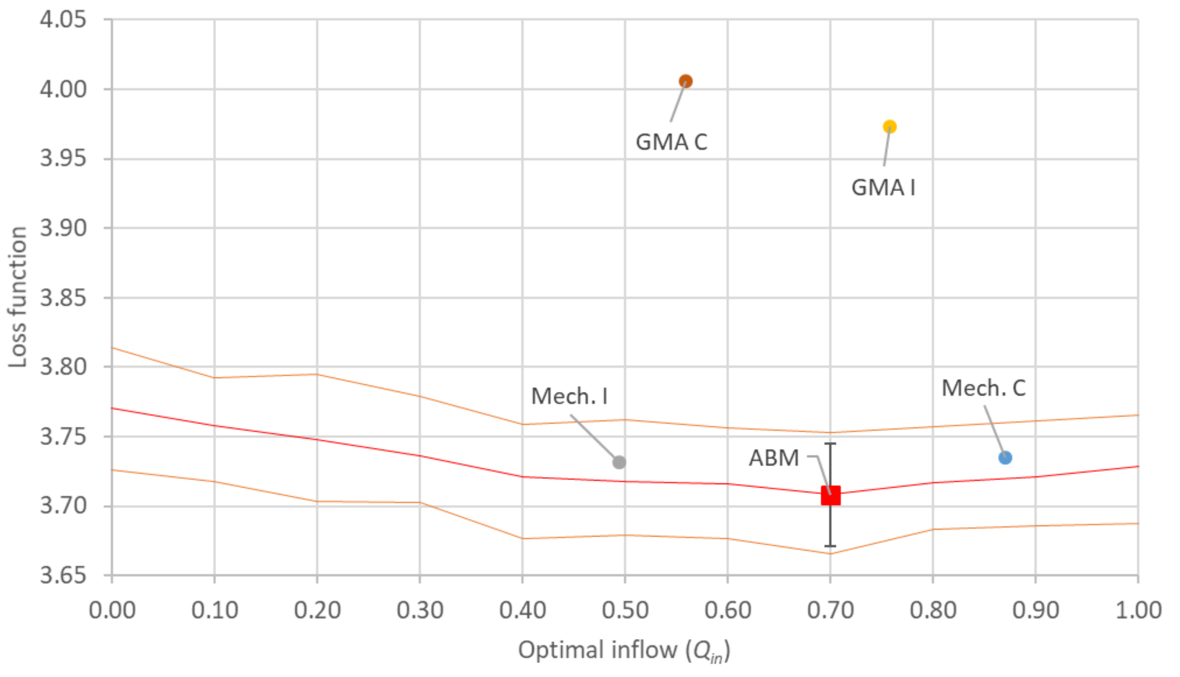

Fig S16. Comparison of effectiveness of different ODE surrogates for solving the metabolic pathway ABM control problem. The red square shows the optimal inflow point and the corresponding mean loss function value as determined for the ABM by a grid search between 0 and 1.0 with a step size of 0.1, where in each step 100 simulations runs were averaged. The red line highlights the mean of each of the 100 simulation runs of the ABM and the orange line the 75% confidence band. Circles denote the predicted optimal inflow and corresponding loss function value for each ODE surrogate. GMA I was the surrogate that best predicted an optimal inflow of substrate closest to the ABM and Mech. I was best at predicting the loss function value of the ABM at the optimal inflow point.

Case 2 – GMA approximation

For the GMA approximation of the metabolic ABM, we used the same stoichiometric matrix as in the mechanistic approximation. On the other hand, the fluxes through each enzyme were approximated to power law terms of all metabolites, and no assumptions were made about which metabolites regulate each of the fluxes. The GMA ODE model is

$$\frac{dX}{dt} = \begin{bmatrix} -1 & 0 & 0 & 0 \\ 1 & -1 & 0 & -1 \\ 0 & 1 & -1 & 0 \\ 0 & 0 & 1 & 0 \\ 0 & 0 & 0 & 1 \end{bmatrix} \cdot \begin{bmatrix} F_A \\ F_E \\ F_I \\ F_O \end{bmatrix} + \begin{bmatrix} Q_{in} \\ 0 \\ 0 \\ 0 \\ 0 \end{bmatrix} - k_{out} \cdot X,$$

$$\begin{aligned} F_A &= p_1 \cdot X_1^{p_5} \cdot X_2^{p_9} \cdot X_3^{p_{13}} \cdot X_4^{p_{17}} \cdot X_5^{p_{21}}, \\ F_E &= p_2 \cdot X_1^{p_6} \cdot X_2^{p_{10}} \cdot X_3^{p_{14}} \cdot X_4^{p_{18}} \cdot X_5^{p_{22}}, \\ F_I &= p_3 \cdot X_1^{p_7} \cdot X_2^{p_{11}} \cdot X_3^{p_{15}} \cdot X_4^{p_{19}} \cdot X_5^{p_{23}}, \\ F_O &= p_4 \cdot X_1^{p_8} \cdot X_2^{p_{12}} \cdot X_3^{p_{16}} \cdot X_4^{p_{20}} \cdot X_5^{p_{24}}, \end{aligned}$$

where $X \in \mathbb{R}^5$ is the vector of the state variables representing the concentrations of S, P, Q, R and T; F_A, F_E, F_I, and F_O are the fluxes through the reactions catalyzed by A, E, I, and O, respectively; Q_{in} is the inflow of S into the system when the system is in continuous mode; k_{out} is the rate of removal of metabolites when the system is in continuous mode; and $p_j \in \mathbb{R}^{24}$ is the vector of kinetic parameters.

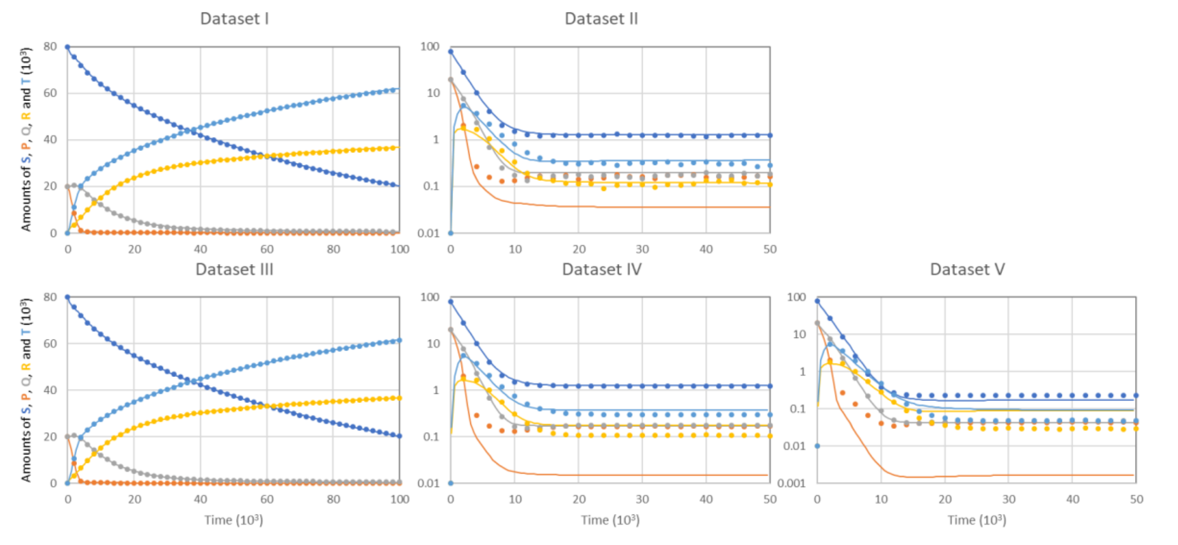

Fig S17. Fit of the GMA approximation (Case 2) to the ABM dataset collections I and C. The GMA approximation (solid line) and corresponding training data (colored markers). The GMA I model fit was obtained with datasets I and II (collection I) and the GMA C model fit was obtained with datasets III-V (collection C). For visualization purposes the datasets were down sampled from 1 point per timestep to 1 point per 2,000 timesteps.

The GMA model was parameterized against the two collections of datasets, I and C (Fig S17), which resulted in two models: GMA I(see file 'MetPw_Case2_GMA.I.m') and GMA C(see file 'MetPw_Case2_GMA.C.m'). These two models were then evaluated for their ability to predict the solution to the control problem (Eq. 15), and the results are shown in Fig S16. Both parameterizations of the GMA model identified similar optima for the inflow of S. The GMA solutions were better than the ones obtained with the mechanistic model, as they are closer to the true value determined for the ABM. Yet, the GMA models were not as good at identifying the

optimal loss function value, and so, overall, the mechanistic approximations performed better than the GMA. Also, the fits of the mechanistic model (Fig S15) were better then the ones obtained for the GMA model (Fig S17).

Case 3 Linear and quadratic approximations in the vicinity of the steady state

We attempted to use the linear and quadratic approximations at the steady state to approximate the metabolic model, even though the model does not have an immediate steady state of interest. We therefore choose the trivial steady state of the dataset I (Fig S14). The steady state of dataset I occurs when the simulation is extended longer than the time horizon shown in Fig S14 and all of S, P, and Q get depleted and all of the mass of the system accumulates in R and T ($[S, P, Q, R, T]_{SS}=[0, 0, 0, 43226, 76794]$). The following ODE models were used:

$$\frac{dX}{dt} = J \cdot \overline{X} + \begin{bmatrix} Q_{in} \\ 0 \\ 0 \\ 0 \\ 0 \end{bmatrix} - k_{out} \cdot X, \qquad \text{(Eq. 18)}$$

$$\frac{dX}{dt} = J \cdot \overline{X} + H \cdot \overline{X2} + \begin{bmatrix} Q_{in} \\ 0 \\ 0 \\ 0 \\ 0 \end{bmatrix} - k_{out} \cdot X, \qquad \text{(Eq. 19)}$$

where $X \in \mathbb{R}^5$ is the vector of the state variables representing the concentrations of S, P, Q, R and T; $\overline{X}=X-X_{SS}$ is the vector of the centered first-order terms; $\overline{X2} = \left[\overline{X}_1 \cdot \overline{X}_1, \overline{X}_1 \cdot \overline{X}_2, \overline{X}_1 \cdot \overline{X}_3, \overline{X}_1 \cdot \overline{X}_4, \overline{X}_1 \cdot \overline{X}_5, \overline{X}_2 \cdot \overline{X}_2, \overline{X}_2 \cdot \overline{X}_3, \overline{X}_2 \cdot \overline{X}_4, \overline{X}_2 \cdot \overline{X}_5, \overline{X}_3 \cdot \overline{X}_3, \overline{X}_3 \cdot \overline{X}_4, \overline{X}_3 \cdot \overline{X}_5, \overline{X}_4 \cdot \overline{X}_4, \overline{X}_4 \cdot \overline{X}_5, \overline{X}_5 \cdot \overline{X}_5\right]^T$ is the vector of centered second-order terms; J is the (5×5) Jacobian matrix of first-order parameters; H is the (5×15) matrix of second-order parameters; Q_{in} is the inflow of S into the system when the system is in continuous mode; and k_{out} is the rate of removal of metabolites when the system is in continuous mode.

Both approximations, Linear (Eq. 18) and Quadratic (Eq. 19), were parameterized against the datasets I and II, which resulted in two models: Linear I (Fig S18, see file 'MetPw_Case3_Linear.I.m'), and Quad I (Fig S19, see file 'MetPw_Case3_Quad.I.m'). These models were then evaluated for their ability to identify the solution to the control problem (Eq. 15), and the results are shown in Fig S20. None of the models were able to predict the optimal inflow of S, as none had a minimum between 0 and 1. The models displayed stiffness, making numerical integration challenging across the entire domain. Linear I could only be simulated for Q_{in} values between 0.45 and 1, and Quad I for values between 0.4 and 1. Given these results, we did not attempt to fit these approximations to datasets III-V.

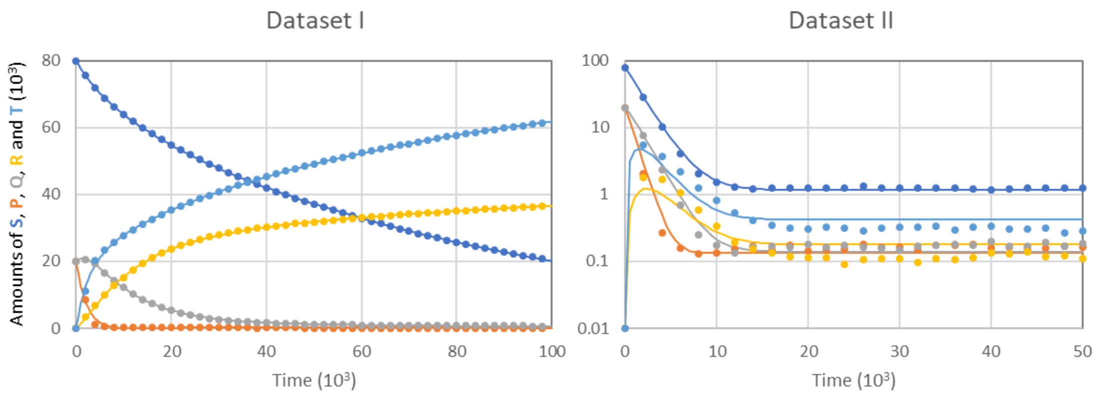

Fig S18. Fit of the linear approximation (Case 3) to the ABM dataset collection I. The linear approximation (solid line) and corresponding training data (colored markers). The Linear I model fit was obtained with datasets I and II (collection I). For visualization purposes the datasets were down sampled from 1 point per timestep to 1 point per 2,000 timesteps.

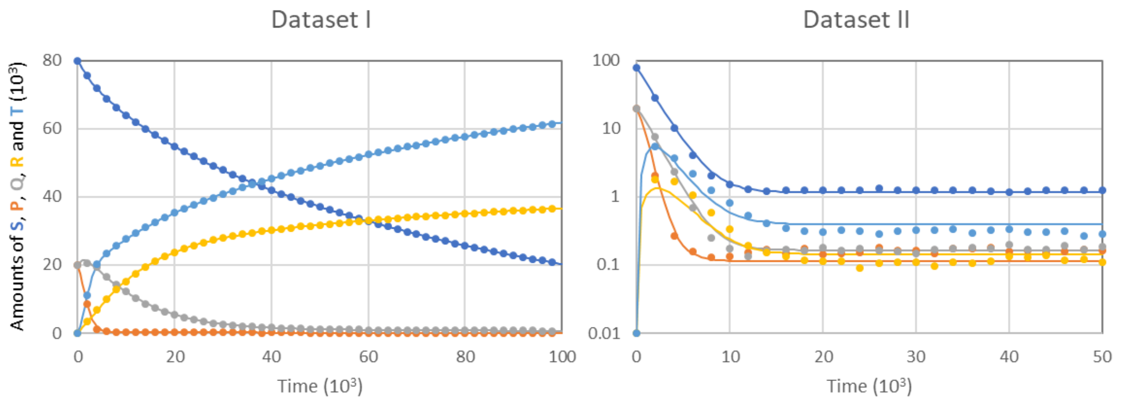

Fig S19. Fit of the quadratic approximation (Case 3) to the ABM dataset collection I. The quadratic approximation (solid line) and corresponding training data (colored markers). The Quad I model fit was obtained with datasets I and II (collection I). For visualization purposes the datasets were down sampled from 1 point per timestep to 1 point per 2,000 timesteps.

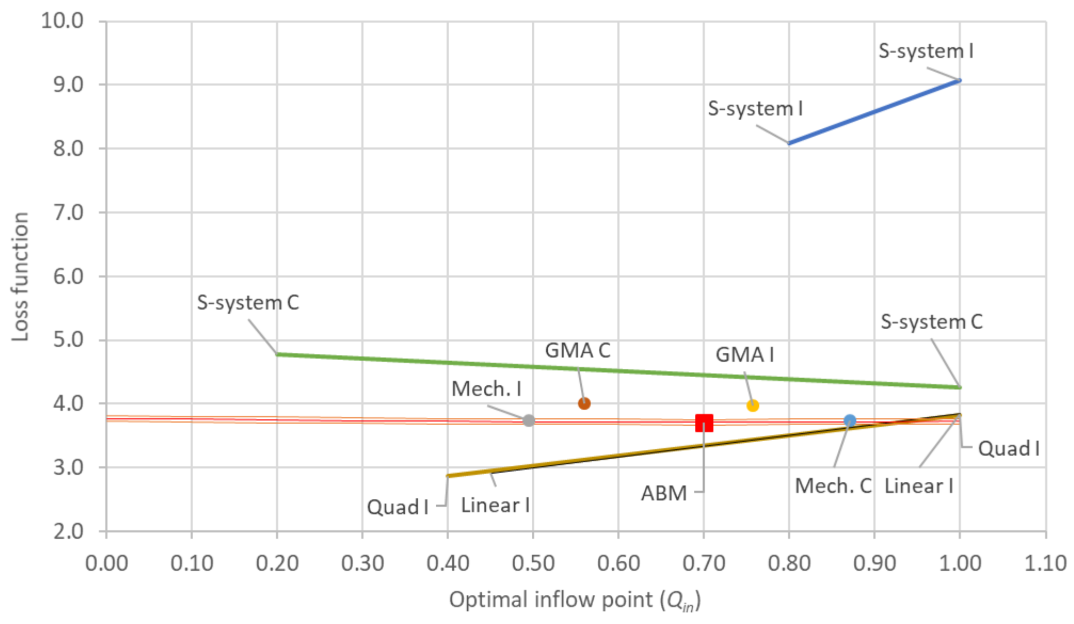

Fig S20. Comparison of effectiveness of different ODE surrogates for solving the metabolic pathway ABM control problem. The red square shows the optimal inflow point and the corresponding mean loss function value as determined for the ABM by a grid search between 0 and 1.0 with a step size of 0.1, where in each step 100 simulations runs were averaged. The red line highlights the mean of each of the 100 simulation runs of the ABM and the orange line the 75% confidence band. Circles denote the predicted optimal inflow and corresponding loss function value for each ODE surrogate. ODE models that did not exhibit a minimum within the 0 to 1.0 domain have their domain of integrability shown with a line. The line depicts the range of loss function values predicted by the approximation. The S-system I performed worst, as it could only be integrated between 0.8 and 1.0, and in that range predicted loss function values between 8 and 9. While S-system C, Quad I, and Linear I, all resulted in models with a larger domain over which they could be integrated, neither had a minimum within their respective domains. GMA I was the ODE surrogate that best predicted an optimal inflow of substrate closest to the ABM and Mech. I best predicted the loss function value of the ABM at the optimal inflow point.

Case 4 The S-system approximation

The S-system approximation of the metabolic ABM is given by the following ODE model:

$$\frac{dX}{dt} = \begin{bmatrix} \alpha_1 \cdot \prod_{j=1}^{5} X_j^{g_{1j}} - \beta_1 \cdot \prod_{j=1}^{5} X_j^{h_{1j}} \\ \alpha_2 \cdot \prod_{j=1}^{5} X_j^{g_{2j}} - \beta_2 \cdot \prod_{j=1}^{5} X_j^{h_{2j}} \\ \alpha_3 \cdot \prod_{j=1}^{5} X_j^{g_{3j}} - \beta_3 \cdot \prod_{j=1}^{5} X_j^{h_{3j}} \\ \alpha_4 \cdot \prod_{j=1}^{5} X_j^{g_{4j}} - \beta_4 \cdot \prod_{j=1}^{5} X_j^{h_{4j}} \\ \alpha_5 \cdot \prod_{j=1}^{5} X_j^{g_{5j}} - \beta_5 \cdot \prod_{j=1}^{5} X_j^{h_{5j}} \end{bmatrix} + \begin{bmatrix} Q_{in} \\ 0 \\ 0 \\ 0 \\ 0 \end{bmatrix} - k_{out} \cdot X, \qquad \text{(Eq. 20)}$$

where $X \in \mathbb{R}^5$ is the vector of the state variables representing the concentrations of S, P, Q, R and T, respectively; Q_{in} the inflow of S into the system when the system is in continuous mode; k_{out} is the rate of removal of metabolites when the system is in continuous mode; $\alpha_i,\ \beta_i \in \mathbb{R}^+$ are the rate constants; $g_{ij},\ h_{ij} \in \mathbb{R}$ are the kinetic orders; and $i, j \in \{1,2,3,4,5\}$.

The S-system approximation was parameterized against the two collections of datasets, I and C (Fig S21), which resulted in two models: S-system I(see file 'MetPw_Case4_Ssystem.I.m') and S-system C (see file 'MetPw_Case4_Ssystem.C.m'). These two models were then evaluated for their ability to identify the solution to the control problem (Eq. 15), and the results are shown in Fig S20. Neither parameterizations of the S-system model were able to identify an optimal inflow of S as neither exhibit a loss function with a minimum between 0 and 1. The worst was S-system I as it was only able to be simulated between 0.8 and 1, while S-system C having been trained with datasets with Q_{in} of 0.2 and 1 was able to be simulated within this range ($0.2<Q_{in}<1$). The failure of the S-system to approximate the metabolic ABM is not surprising as this ABM was specifically designed to be challenging for the S-system method. This ABM's processes are Michaelian, and S starts at high values relative to the Km of enzyme A. This poses an issue to power laws as these functions are only good at approximating Michaelis-Menten processes in small regions (Fig S22), which is likely what to be found if we were dealing with a real *in vivo* metabolic pathway. However, this same argument applies to the GMA approximation and this approximation did perform well. Additionally, the S-system also has problems approximating nodes with several processes coming in or out, as all processes in and all processes out of a node are approximated to single power laws. This is in contrast to the GMA approximation that has a power law function for each process and therefore does not suffer from this issue. Most likely, these two problems together prevented the S-system from producing good approximations to this ABM.

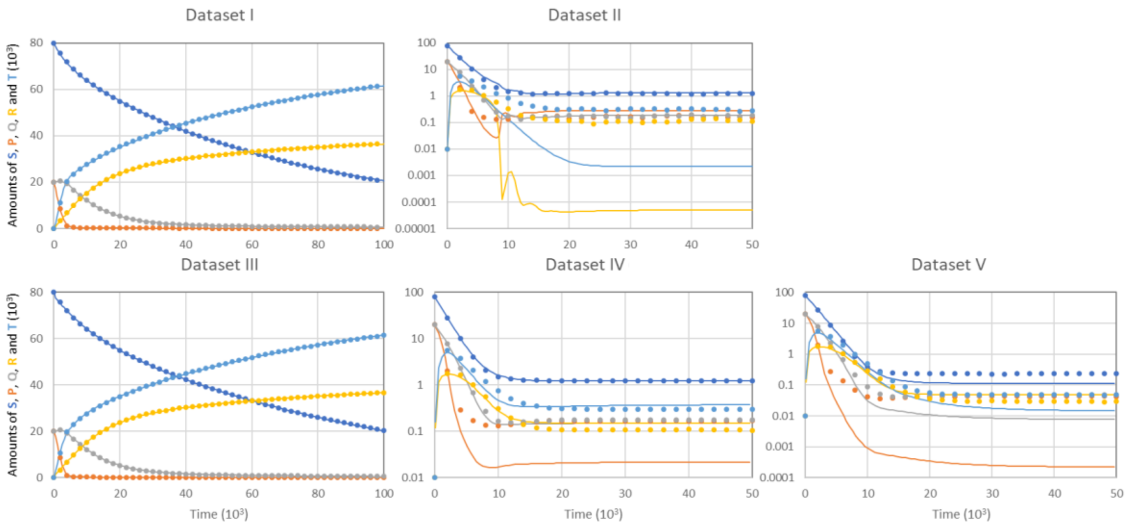

Fig S21. Fit of the S-system approximation (Case 4) to the ABM dataset collections I and C. The S-system approximation (solid line) and corresponding training data (colored markers). The S-system I model fit was obtained with datasets I and II (collection I) and the S-system C model fit was obtained with datasets III-V (collection C). For visualization purposes the datasets were down sampled from 1 point per timestep to 1 point per 2,000 timesteps.

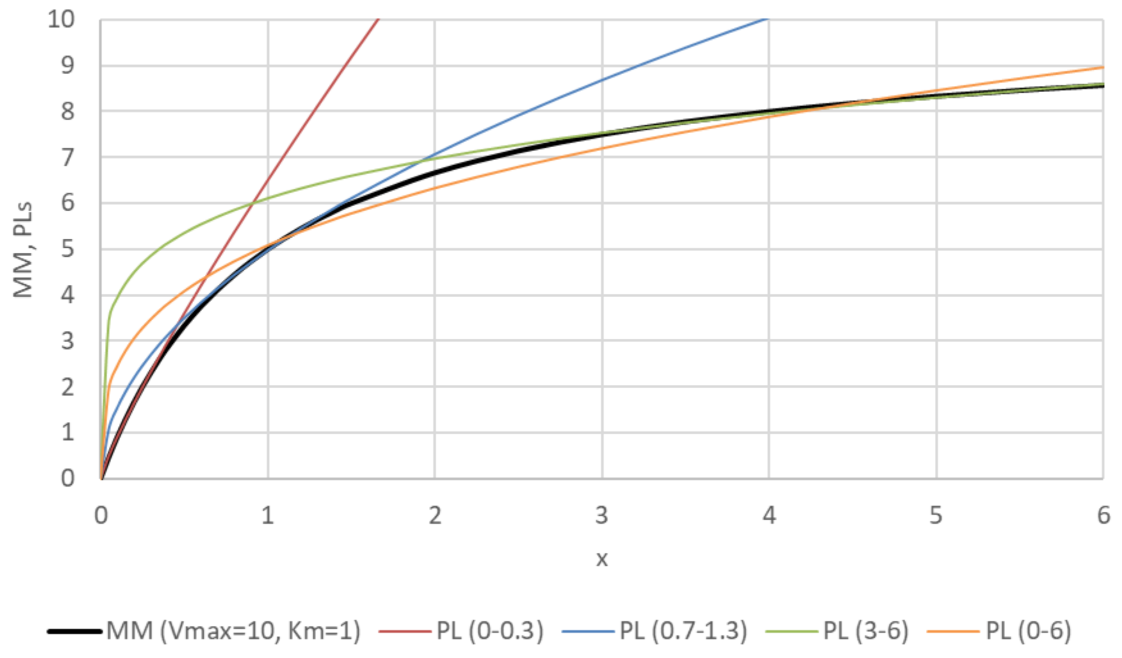

Fig S22. Comparison of a Michaelis-Menten curve and 4 power laws fitted against different regions of the Michaelis-Menten curve. The solid black line shows a Michaelis-Menten (MM) function ($MM(x) = x \cdot Vmax/(x + Km)$) with a Vmax of 10 and a Km of 1. The red power law (PL) function ($PL(x) = \alpha \cdot x^{g}$) was fitted only against the region of the MM function between 0<x<0.3, where it agrees well but diverges for x>0.3. The blue power law was fitted in the region of the Km, 0.7<x<1.3, where it agrees well with the MM function, but diverges everywhere else. The green power law was fitted only against the region of the MM function between 3<x<6, where it agrees well but diverges for x<3. In contrast, the orange power law was fitted against the entire domain of the MM function shown, 0<x<6, and does not appropriately approximate the MM function anywhere except in two points around 1.1 and 4.6. Additionally, above 6 (x>6) all power laws will keep increasing, while the MM function has an asymptotic limit given by Vmax (10 in this example).

References

1. Wilensky U. NetLogo wolf sheep predation model. Center for Connected Learning and Computer-Based Modeling, Northwestern University, Evanston, IL. 1997. Available: http://ccl.northwestern.edu/netlogo/models/WolfSheepPredation

2. Wilensky U. NetLogo. Center for Connected Learning and Computer-Based Modeling, Northwestern University, Evanston, IL. 1999. Available: http://ccl.northwestern.edu/netlogo/

3. Olivença DV, Davis JD, Voit EO. Comparison Between Lotka-Volterra and Multivariate Autoregressive Models of Ecological Interaction Systems. 2021 Oct p. 2021.10.07.463461. doi:10.1101/2021.10.07.463461

4. Voit EO, Davis JD, Olivença DV. Inference and Validation of the Structure of Lotka-Volterra Models. 2021 Aug p. 2021.08.14.456346. doi:10.1101/2021.08.14.456346

5. Stein RR, Bucci V, Toussaint NC, Buffie CG, Rätsch G, Pamer EG, et al. Ecological Modeling from Time-Series Inference: Insight into Dynamics and Stability of Intestinal Microbiota. PLOS Comput Biol. 2013;9: e1003388. doi:10.1371/journal.pcbi.1003388

6. Tibshirani R. Regression Shrinkage and Selection via the Lasso. J R Stat Soc Ser B Methodol. 1996;58: 267–288.
